# Perspectives on Usage of Functional Nanomaterials in Antimicrobial Therapy for Antibiotic-Resistant Bacterial Infections

**DOI:** 10.1021/acsomega.3c00110

**Published:** 2023-04-06

**Authors:** Arun Karnwal, Gaurav Kumar, Gaurav Pant, Kaizar Hossain, Akil Ahmad, Mohammed B. Alshammari

**Affiliations:** † Department of Microbiology, School of Bioengineering & Biosciences, Lovely Professional University, Phagwara, Punjab 144411, India; ‡ Department of Microbiology, Graphic Era (Deemed to be University), Dehradun, Uttarakhand 248002, India; § Department of Environmental Science, Asutosh College, University of Calcutta, 92, Shyama Prasad Mukherjee Road, Bhowanipore, Kolkata 700026, West Bengal, India; ∥ Department of Chemistry, College of Science and Humanities in Al-Kharj, Prince Sattam Bin Abdulaziz University, Al-Kharj 11942, Saudi Arabia

## Abstract

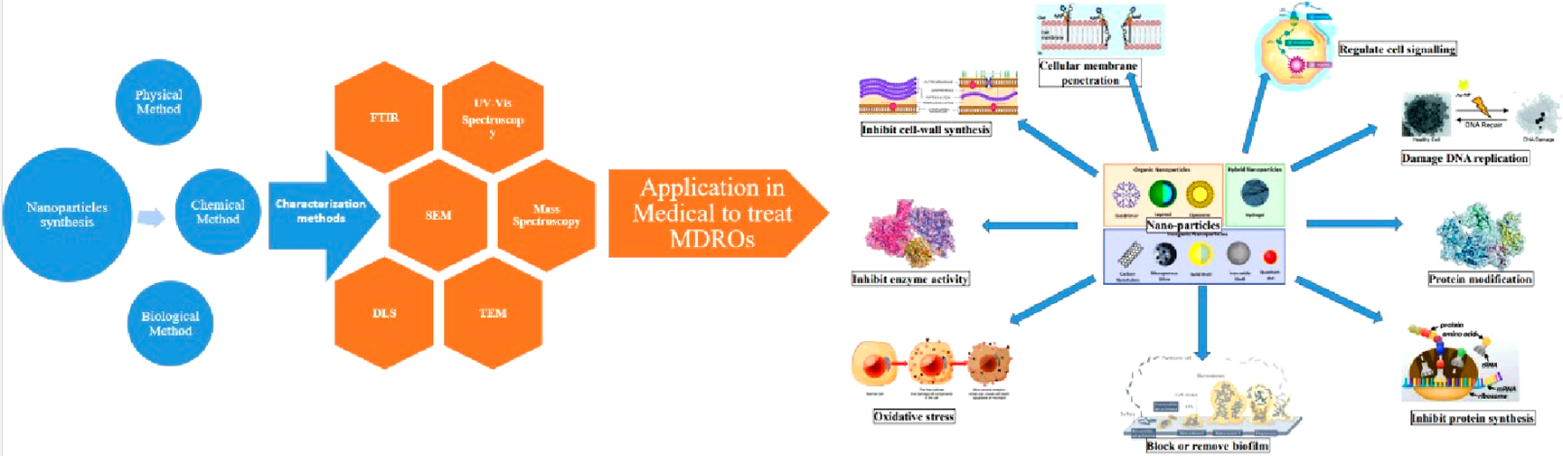

The clinical applications of nanotechnology are emerging as widely popular, particularly as a potential treatment approach for infectious diseases. Diseases associated with multiple drug-resistant organisms (MDROs) are a global concern of morbidity and mortality. The prevalence of infections caused by antibiotic-resistant bacterial strains has increased the urgency associated with researching and developing novel bactericidal medicines or unorthodox methods capable of combating antimicrobial resistance. Nanomaterial-based treatments are promising for treating severe bacterial infections because they bypass antibiotic resistance mechanisms. Nanomaterial-based approaches, especially those that do not rely on small-molecule antimicrobials, display potential since they can bypass drug-resistant bacteria systems. Nanoparticles (NPs) are small enough to pass through the cell membranes of pathogenic bacteria and interfere with essential molecular pathways. They can also target biofilms and eliminate infections that have proven difficult to treat. In this review, we described the antibacterial mechanisms of NPs against bacteria and the parameters involved in targeting established antibiotic resistance and biofilms. Finally, yet importantly, we talked about NPs and the various ways they can be utilized, including as delivery methods, intrinsic antimicrobials, or a mixture.

## 1 Introduction

Bacteria were the oldest living things to be identified on Earth, and throughout billions of years, they have evolved to become extraordinarily adaptable to the environment.^1^ During the 20th century, the discovery of antibiotics was considered one of the most important medical discoveries ever made. It commenced with Salvarsan, among the first drugs to cure syphilis without harming sufferers.^2^ Although, research on antibiotics did not begin until 1928 when Alexander Fleming discovered penicillin by accident. This research reached its pinnacle in the 1950s and 1960s, which emerged as the “golden period” of antibiotics study. During 1930 and 1962, over 20 new antimicrobial classes were discovered; however, new bacteria strains emerged that were resistant to existing antibiotics, making it even more difficult for drug companies to find new compounds that have antibacterial activity.^2,3^ The development of antibiotic resistance in bacteria has led to the difficult problem of treating resistant infections. The emergence of bacteria that are resistant to multiple drugs is a global problem that is increasing the risk of morbidity and mortality among infected people and having a negative impact on the clinical outcome of a diverse range of patients like those admitted into the ICU, recently operated on or undergoing operation, organ transplant, or treatment for cancer.^4,5^ Antibiotic resistance was identified as a global problem in a report published in 2017 by the WHO Global Antimicrobial Surveillance System. The anticipated cost of treating infections that are resistant to antibiotics is high (about US$50,000 per individual), and the annual cost to society is estimated to be US$20 billion.^6^ This already serious threat to public health is made even worse because there are so few novel therapies in development for antibiotics and the widespread use of antibiotics, some of which are even inappropriately prescribed. Planktonic bacteria, often known as free-floating bacteria, are major contributors to various health risks, including sepsis.^7−9^ Infections linked to planktonic bacteria pose serious risks and are fast becoming more difficult to treat due to increased rates of antibiotic resistance that patients have acquired over time. This difficulty is compounded during biofilm production by bacteria, which are linked to recurrent and persistent bacterial infections. Biofilms complicate bacterial infection treatment.^10,11^ The ability of bacteria to hide under biofilms makes it significantly more complicated to manage a broad array of diseases, including infectious endocarditis, osteomyelitis, and chronic wounds. Biofilm-associated antibiotic resistance differs from acquired resistance, but it can complicate the treatment used in therapy.^12,13^ Bacterial cells are capable of producing extracellular polymeric substances (EPS), which have the potential to operate as a barrier against immunological responses from the host and certain traditional antimicrobial treatments. In addition, biofilms show various altered phenotypes contributing to resistance to many commonly used antibiotics. These altered phenotypes are included spatial and chemical heterogeneities, the presence of persister cells, and slow growth rates.^14−16^ Antibiotics are the primary therapeutic method used at the moment for treating biofilm and planktonic infections. They focus on processes critical for the development and/or survival of bacteria, such as the formation of DNA, RNA, or essential proteins; cell wall formation and regulation; and essential protein production.^3,17,18^ Most antibiotics are produced from compounds used for billions of years by different microbes to fight against each other. Many antibiotics are derived from these products. In the course of this warfare, offensive molecules have evolved, resulting in the development of defensive reactions; bacteria have evolved resistance to several commonly used antibiotics, i.e., MRSA.^19,20^ In order to eradicate MRSA, it may be necessary to utilize various antibiotic agents, high dosages of antibiotics or medications considered a “last resort.” When bacteria are located in biofilms, biofilm-associated resistance creates a potential factor, making it necessary to remove the biofilm physically, for example, using rigorous exfoliation, sometimes accompanied by large dosages of antibiotics.^21,22^ This adds to the difficulty of providing effective treatment for the infection. These tactics might result in therapies that are drawn out and expensive, with the potential for unfavorable side effects and a lack of clarity regarding the final outcome. Nanomaterials utilize antibacterial modes that bacteria have never seen before and, thus, have no defenses against these new antimicrobial materials.^7^ Recent developments in systems based on nanomaterials have opened up new avenues for combating multidrug-resistant infections in planktonic and biofilm forms of infection. These systems can either operate as inherent therapies or as nanocarriers for antimicrobial drugs. The therapeutic activity of nanomaterials is influenced in several ways by their one-of-a-kind physicochemical features, such as their size, shape, and surface chemistry.^11^ The shapes and sizes of various nanomaterials are analogous to bacterial biomolecules, which allow a range of interactions that can be managed using surface modification. Antibacterial nanoparticles demand substantial surface-to-volume ratios and multivalent interactions. Nanomaterials can circumvent the resistance mechanisms that are already in place, and they may be less likely to select for resistance than traditional antibiotics.^23^ In addition, nanoparticles can wipe off bacteria present in biofilms. Together, these evidence suggests that nanotechnology can be used as a new resource in developing techniques to treat MDR infections.^24,25^ In this review, we explore the potential applications of nanomaterials in the fight against multidrug-resistant bacterial diseases. We explore the features and design components that result in therapeutic efficacy, thereby providing insight into how nanomaterials could be adjusted to improve action against biofilm and planktonic bacteria. In conclusion, we discuss the current state of the clinical development of antibacterial nanomaterials.

## 2 Metal Nanoparticles

The size, shape, roughness, and surface energy of nanoparticles are some of the most important properties to be considered during their application. Among inorganic nanoparticles, metal-based nanoparticles are the most widely used and offer the opportunity to tackle antibiotic resistance issues. In addition to being effective against bacteria that have evolved resistance to conventional antibiotics, their unique modes of action also target several macromolecules, making it more difficult for resistant strains to evolve.^38,82^ Numerous methods can be utilized in order to characterize nanoparticles composed of metal. These approaches provide helpful information regarding the particles’ shape, physical and chemical properties, and electric properties, which are essential for the in-vivo particles’ activity.^24−26^

### 2.1 Action of Metal-Based Nanoparticle

In the presence of metal nanoparticles, bacteria exhibit certain behaviors, which their unique properties can explain. It is essential to comprehend the differences between Gram-negative and Gram-positive cell wall structures since the main cytotoxic action produced by antibacterial agents in bacteria occurs by close interaction with the cell wall.^26^ Gram-negative and Gram-positive bacteria have a negatively charged surface due to their polar lipid bilayers. Gram-positive bacteria contain a thick coating of peptidoglycan made up of N-acetylglucosamine (NAG) and N-acetylmuramic acid (NAM) that cross-link each other, making a strong network.^27^ Furthermore, the majority of Gram-positive bacteria have negatively charged teichoic acids. These acids have significant quantities of negatively charged phosphate groups.

On the other hand, Gram-negative bacteria have a structure that is just somewhat more complicated.^28,29^ Gram-negative bacteria have lipopolysaccharides (LPS) as an outer layer, which add negative charge surface quality to the cell envelope. This is in conclusion to the thin peptidoglycan layer that covers the outer surface of their cell walls.

Electrostatic forces cause negatively charged bacterial cell walls to attract positively charged nanoparticles to their surface. This happens because positively charged nanoparticles are attracted to negatively charged surfaces. In contrast, positively charged metal-based nanoparticles form a strong bond with membranes, which leads to the rupture of cell walls and, as a result, increases the permeability of the membranes.^30−32^ In addition, nanoparticles can release metal ions from the extracellular space. These ions can then enter the cell and wreak havoc on the biological processes. Both metal ions and nanoparticles can potentially stimulate the creation of reactive oxygen species within the cell (ROS). The oxidative stress produced results in the oxidation of glutathione, inhibiting the antioxidant defense mechanism bacteria have against ROS.^33^ The metal ions are then free to interact with the structures of the cell (such as proteins, membranes, and DNA), which disrupts the cell’s functioning. Metal ions can form strong covalent bonds with the nitrogen, oxygen, or sulfur atoms commonly found in organic compounds and biomolecules. As a result of the largely nonspecific nature of the link between metal ions and biomolecules, nanoparticles based on metal almost always display a broad spectrum of activity.^7^

### 2.2 Metal Nanoparticle Synthesis

**Figure 1:**
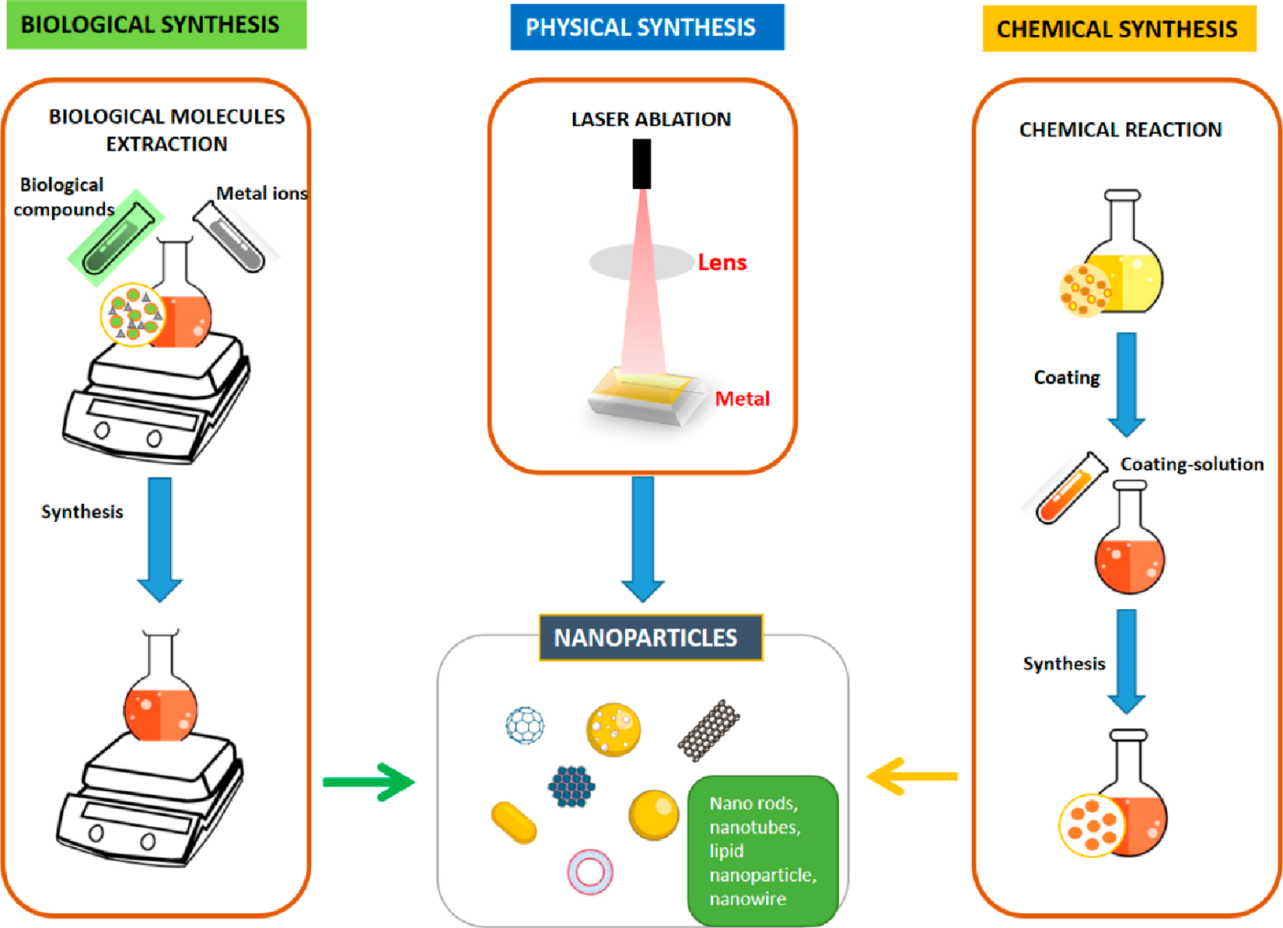
Different approaches used for the synthesis of nanoparticles.^40^

Metal nanoparticles are not new. Some microbes have been shown to naturally produce metal-based nanoparticles as a method for the detoxifying of heavy metals, and this process has been documented.^34^ However, the adaptability of this technique has only been detailed in recent decades, and since then, metal-based nanoparticles have seen widespread application in the production of cosmetics and textiles.^35^ The adaptability of these substances has attracted the scientific world’s attention, which has continued on a never-ending pursuit of novel formulations, applications, and synthesis techniques. Although research has recently extended to less-common metals, silver, gold, copper, iron, and zinc are the most extensively used materials in metal-based nanoparticles.^36−38^ It is anticipated that transition metals will be the ideal choice for producing metal-based nanoparticles. This is because transition metals have partially filled d-orbitals, which makes them more redox-active. This property makes it easy for transition metal nanoparticles to aggregate with one another.^39^ Different methods of synthesis that have been developed can be arranged into three categories (Figure 1): physical, chemical, and biological methods.

When using physical methods, a top-down strategy is taken, beginning with a large piece of metal that is fragmented into small parts by physical action into progressively smaller fragments. Because this method produces nanoparticles with a somewhat scattered size distribution, it is not the most acceptable for synthesizing metal-based nanoparticles. Their size determines the activity of metal-based nanoparticles, so the most appropriate method would be one that produces nanoparticles with a more uniform size. On the other hand, bottom-up strategies are utilized with chemical procedures that use chemical solvents and biological approaches, which are focused on eco-friendly processes employing various types of microorganisms. Both of these types of methods involve the use of chemical solvents (Table 1).^40^

**Table 1:** Nanoparticle Properties and Modes of Action against Multidrug-Resistant (Mdr) Microorganisms^2,8,19,25,28,30,35,41−44^

size	antibacterial mechanisms	nanoparticles (NPs)	targeted bacteria and antibiotic resistance	factors affecting antimicrobial activity/toxicity
1–100 nm	cell wall perforations, bacterial membrane breakdown, ATPase activity reduction, respiratory chain disruption, and loss of membrane potential	AuNPs	MRSA *S. aureus*	size and roughness
1–100 nm	modification in nucleotides, inhibit protein synthesis by affecting ribosome, lipid and protein damage, boost cellular membrane permeability for many solutes, inhibit bacterial cellular membrane synthesis and cell wall synthesis, blocking ETS (electron transport chain) in bacteria, ROS production, and oxidative stress	AgNPs	carbapenem-resistant Enterobacteriaceae (CRE) and *P. aeruginosa*, B-resistant *A. baumannii*, *K. pneumoniae*, *Pseudomonas aeruginosa*, ESBL *E. coli*	size and shape
2–350 nm	modification in nucleotides, DNA damage, lipid and protein damage, boost cellular membrane permeability for many solutes, ROS production, and oxidative stress	CuNPs	*A. baumannii*, MDR *E. coli*	size and concentration
20–400 nm	breakdown bacterial cell wall using ROS	SiNPs	MRSA *S. aureus*	size, shape, and stability
10–100 nm	breakdown bacterial cell wall using ROS	AlNPs	MDR *E. coli*	
1–100 nm	oxidative stress	iron-oxide NPs	*K. pneumoniae*, MRSA *S. aureus*, MDR *E. coli*	enhanced chemical reaction, able to aggregate
10–100 nm	lipid and protein damage, boost cellular membrane permeability for many solutes, ROS production, and oxidative stress	ZnO NPs	*E. aerogenes*, *E. coli*, *K. oxytoca*, *K. pneumoniae*, MRSA *S. aureus*, extended-spectrum beta-lactamase *E. coli*, *K. pneumoniae*	size and concentration
30–45 nm	ROS production, oxidative stress, and adhesion on the cellular surface	TiO_2_ NPs	*Enterococcus faecium*, *S. aureus*, *P. aeruginosa*, *E. coli*	size, shape, and crystal structure
15–100 nm	peroxidation of lipid and ROS production	MgO NPs	*E. coli*, *S. aureus*	size, concentration, and pH

### 2.3 Characterization of Nanomaterials

Size and form are two essential characteristics analyzed in the NP characterization process. Researchers could also analyze the surface chemistry by measuring the size and distribution, degree of aggregation, surface charge, and surface area. Size, size distribution, and the presence of organic ligands on the surface of the particles are all factors that can influence other aspects of the NPs and their potential uses. Moreover, the NPs’ crystal structure and chemical build are carefully examined as a preliminary step after nanoparticle synthesis. It is of the utmost importance to comprehensively characterize the nanomaterials created in various methods.^8,45^

These methods may be used alone or together to examine the property (Table 2). There are techniques based on microscopy that can provide information on the size, morphology, and crystal structure of nanomaterials.^46^ Some examples of these techniques include transmission electron-microscopy (TEM), high-resolution transmission electron-microscopy (HRTEM), and atomic force microscopy (AFM). Some methods, like the magnetic approaches, are specifically geared for working with particular categories of materials.^47^ Superconducting quantum interference devices (SQUID), vibrating sample magnetometer (VSM), ferromagnetic resonance (FMR), and X-ray magnetic circular dichroism (XMCD) are a few examples of the methods that fall under this category.^48−50^ Numerous more techniques, including X-ray, spectroscopy, and scattering techniques, offer additional data on the structure, elemental composition, optical characteristics, and other standard and more specialized physical features of the nanoparticle samples as mentioned in Table 2.^38,51^

**Table 2:** Determining Parameters and Characterizing Methods Used for Nanoparticle Characterizations

property characterized	approaches used for characterization
size (structural properties)	EPLS, TRPS, NMR, MALDI, UV–vis, ICP-MS, DCS, EXAFS, AFM, DLS, XRD, SEM, TEM, HRTEM, and magnetic susceptibility
size distribution	FMR, ICP-MS, NTA, SAXS, DLS, DCS, TRPS, DTA, SEM, and superparamagnetic relaxometry
shape	3D-tomography, FMR, EPLS, AFM, TEM, and HRTEM
crystal structure	EXAFS, XRD, electron diffraction, STEM, and HRTEM
chemical state and oxidation state	XPS, EELS, XAS, and Mössbauer spectroscopy
elemental and chemical composition	MFM, SEM-EDX, ICP-OES, NMR, ICP-MS, LEIS, XRD, and XPS
surface charge	EPM and zeta-potential
surface area and specific surface area	liquid NMR and BET
growth kinetics	liquid-TEM, cryo-TEM, TEM, NMR, and SAXS
concentration	DCS, PTA, RMM-MEMS, UV–vis, and ICP-MS
agglomeration state	TEM, cryo-TEM, SEM, UV–vis, DCS, zeta-potential, and DLS
density	RMM-MEMS and DCS
single particle properties	liquid TEM, HRTEM, MFM, and Sp-ICP-MS
3D visualization	SEM, AFM, and 3D-tomography
NPs distribution in matrices/supports	AFM, TEM, and SEM
abnormalities in the structure	BSD and HRTEME
NPs detection	EBSD, SEM, STEM, TEM, and magnetic-susceptibility
optical properties	EELS-STEM and UV–vis-NIR
magnetic properties	XMCD, FMR, MFM, Mössbauer spectroscopy, VSM, and SQUID

## 3 Nanozymes

Nanozymes can perform various enzyme-like functions in different areas, such as regulating biomolecular and cellular pathways, cleaving proteins or poly nucleic acids, modulating oxidative balance, and performing site-specific cleavage of prodrugs.^52,53^ These functions make nanozymes useful in different applications, including therapeutics, regenerative medicine, diagnostics, and preservation. Endogenous enzymes in the cell catalyze metabolic events and produce hazardous ROS that potentially kills the bacterial cell membranes and intracellular components by oxidation.^54^ However, natural enzymes have their limits; thus, synthetic nanozymes are increasingly employed in antibacterial therapy as promising alternatives with an antibiotic-free environment. Furthermore, nanozymes are not susceptible to acquiring resistance by bacteria because of their biocompatibility and excellent cellular membrane permeability. Moreover, nanozymes can be engineered with specialized catalytic activity to remove biofilms efficiently.^55^

### 3.1 Metal-Based Nanozymes

Noble-metal-based nanozymes have been found to have the potent catalytic capability. In an earlier study, Zheng et al.^56^ used mercapto-pyrimidine-conjugated Au-nanoclusters to target superbugs and found that the positively charged nanozymes adhered easily to bacterial surfaces and caused cell membrane damage. The nanozymes triggered the generation of intracellular ROS in bacterial cells that accelerate wound healing and kill >99% of bacteria due to the oxidase and peroxidase-like activity. Similarly, Zhang et al.^57^ evaluated the antibacterial potency of bimetallic PtCu alloy NPs, which also had ferroxidase-like and peroxidase-like activity in a high pH solution, and detected Fe_2+_. Cai et al.^58^ synthesized core–shell Pd@Ir bimetallic nanomaterials with morphology-dependent bactericidal activity. In another report,^59^ Cu-based nanozymes having POD-like characteristics were also developed, and hydrogel-based nanozymes embedded with Cu were found to accelerate wound healing by stimulating angio-genesis and collagen-deposition with H_2_O_2_ assistance.

### 3.2 Metal Oxide/Sulfide-Based Nanozymes

Cerium-oxide (CeO_2_) NPs possess high peroxidase-like activity, which is attributed to the reversible redox switch between Ce^4+^ and Ce^3+^ ions. When CeO is combined with H_2_O_2_, it generates reactive oxygen species (ROS) due to its effective peroxidase-like activity.^60^ Various sizes and shapes of nanoceria exhibit multiple enzymatic activities, like oxidase (OXD), peroxidase (POD), catalase (CAT), and superoxide dismutase (SOD) due to their high redox potential, smooth oxygen diffusion, and surface-rich oxygen vacancies. In a study by Luo et al.,^61^ an electrospun nanofibrous membrane composed of imidazolium-type-poly(ionic liquid) (PIL) and Ce^4+^ (PIL-Ce) was developed, which exhibited DNase-like catalytic properties and accelerated wound healing in a Methicillin-resistant *Staphylococcus aureus* infected mice model. PIL-Ce also demonstrated high antibacterial potential and disintegrated resistant genes to prevent drug resistance. Additionally, Gao et al.^62^ synthesized nanoiron sulfide particles using a garlic-derived natural-organo-sulfur compound, which exhibited a broad-spectrum antimicrobial effect toward resistant bacterial pathogens. Nanoiron sulfide acts as a nanozyme with CAT-like and POD-like activities, catalyzing the H_2_O_2_ oxidation to produce highly toxic hydrogen-polysulfide and resulting in 500× enhanced antimicrobial potential against resistant bacterial pathogens.^63^ As an added advantage, these nanozymes could help to improve the healing process and fight against biofilms on human dental caries.

### 3.3 Carbon-Based Nanozymes

Carbon-based nanomaterials have gained popularity in biomedicine because of their desirable physical and chemical characteristics, biocompatibility, and ability to mimic various enzymes. Such materials have great mechanical qualities and can be used as wound dressings; examples are fullerene, graphene and its derivatives, carbon nitride, carbon dots (CDs), and carbon nanotubes (CNTs).^64^ The peroxidase-like activity of a series of oxidized carbon nanotubes (o-CNTs) produced by Wang et al.^65^ was exceptional throughout a broad pH range. The carbonyl-group on the oxidized carbon nanotube surface served as an active catalytic core, with the hydroxyl and carboxyl groups serving as competing sites. The researchers^65^ developed o-CNTs-BrPE to lessen the limiting impact of the carboxyl-group, which has a stronger tendency to suppress catalytic activity than the hydroxyl group. o-CNTs-BrPE showed strong POD-like action, catalyzing the conversion of H_2_O_2_ to OH, which led to bacterial elimination and tissue protection from purulent inflammation and bacterial-induced edema by lowering the number of competing sites.

### 3.4 Transition Metal Dichalcogenide (TMDC) as Nanozymes

Transition metal dichalcogenides (TMDCs) are a class of 2D materials with promising antibacterial properties due to their enzyme-like properties and large surface area. Earlier, MoS_2_/rGO (a defect-rich adhesive) vertical heterostructure was developed, demonstrating exceptional antibacterial activity because of surface defects and OXD-like, CAT-like, and POD-like activity.^66^ Another study^67^ found that flower-shaped MoS_2_ nanozymes with rough surfaces and active edges exhibited superior antibacterial efficacy compared to other MoS2 nanozymes. Additionally, TMDC-based NPs can be photoactivated to enhance enzymatic activity, such as in Cu_2_MoS_4_ nanozymes which exhibited remarkable antibacterial activity against MDR *S. aureus* and *E. coli* upon irradiation with near-infrared light.^68^ A charge-tunable MoS_2_ nanozyme was also developed and light-modulated for charge reversal on the surface and enzymatic activation upon varying pH levels. AgNPs have also shown broad-spectrum antibacterial properties as they mimic enzymes like POD, OXD, CAT, and SOD. A hybrid Fe3O4@MoS_2_–Ag nanozyme was constructed and demonstrated significant antibacterial activity through the near-infrared-light-activated photothermal effect, Ag^+^ ions leakage, and POD-like activity, resulting in ROS production. The magnetic property of Fe_3_O_4_ allowed for the recycling of the nanozyme.^69^

### 3.5 Metal–Organic Framework (MOF) as Nanozymes

Metal–organic frameworks (MOFs) are a type of nanomaterial that consists of metal nodes and organic bridging linkages to form 3D structures with tailorable pore widths, high surface areas, and a wide variety of porous architectures. The appropriate arrangement of active catalytic sites in a biocompatible MOF allows it to stabilize endogenous enzymes and operate as catalytic sites with high enzyme-like activity.^70,71^ Zhang et al.^71^ produced a MOF-based nanozyme that resembles peroxidase (POD) and has surfaces resembling pseudopodia to improve bacterial trapping. The MOF’s metal nodes function as the active centers, while the nanoscale cavities play the role of binding pockets. The microenvironment around the active site helps to increase and activate the substrate molecules, which in turn inhibit bacterial growth. Another research group^70^ developed a nanozyme based on Au-doped MOF/Ce that exhibited DNase and POD-like activity to eliminate biofilms. The DNase-like activity of the MOF hydrolyzes extrinsic DNA and biofilm constituents, while the POD-like function of the MOF inhibits biofilm-forming bacteria.

### 3.6 Single Atom Nanozymes (SANs)

SANs have distinct catalytic regions and can be used for diverse applications. Unlike conventional nanozymes, SANs have an even distribution of metal centers, which maximizes the active sites and increases their catalytic activity and specificity.^72^ SANs like Pt–Cu, Pt/CeO_2_, M-N5, and M-N_4_ (M = Fe, Co, Zn, etc.) have been developed and show different enzyme-mimicking characteristics, such as glutathione peroxidase (GPx-like), CAT, SOD, and POD-like activities, with application in organic pollutant degradation, therapeutic diagnosis, antibacteria, and anti-inflammation.^73,74^ Researchers have synthesized different types of SANs with unique properties. In another study, Shi et al.^75^ developed single-iron-atom nanocatalysts by embedding them in N-doped amorphous carbon, demonstrating outstanding antimicrobial properties toward Gram-negative and Gram-positive bacteria. Liu et al.^76^ developed ZIF-8 (zeolitic-imidazolate framework) derived carbon nanomaterial with effective POD-like activity that suppressed bacterial development and improved in vivo wound healing and disinfection in infected wounds. Furthermore, Huang et al.^73^ reported SANs with carbon nano frame-confined FeN_5_ active centers, which catalytically behaved like the axial ligand-coordinated scheme of cytochrome P450 and exhibited the maximum oxidase-like activity with versatile antibacterial applications.

## 4 Antimicrobial Mechanism of Nanoparticles

### 4.1 Mechanisms against Planktonic Bacteria

Nanomaterials have a diverse range of sizes and shapes, allowing them to target bacteria in a way that no other material can (Table 1). Nanomaterials have the potential to kill bacteria through a variety of processes, such as inflicting direct damage to the cell wall or membrane, affixing themselves to cellular elements, and producing reactive oxygen species (ROS).^77−79^ Most antimicrobial compounds disturb intracellular biochemical pathways or attack microbial cell-wall or membranes. Nanomaterials can attack these cell components and characteristics and provide advantages against antimicrobial drugs to fight against antibiotic-resistant pathogens. In addition, nanomaterials have the potential to act as nanocarriers, which would allow for the delivery of medicinal substances. Nanomaterials’ methods directly result from the one-of-a-kind physicochemical features they possess, particularly their multivalent interactions with bacterial cells.^80,81^ At the interfaces of nanomaterials and bacteria, several forces, including hydrophobic interactions, receptor–ligand interactions, electrostatic attractions, and van der Waals forces, all play essential roles.

### 4.2 Damage to Cellular Contents

Bacterial function and survival rely on cell homeostasis and intracellular signaling. It is possible to create nanomaterials to interfere with these processes, ultimately resulting in the cell’s death.^37^ These disturbances include changes in the expression of genes, alterations in protein synthesis, and damage to DNA. For example, pyrimidine-capped AuNPs (Au–DAPT) were produced by functionalizing AuNPs with an analogue of 2-pyrimidin-ethiol (4,6-diamino-2-pyrimidin-ethiol) present in *E. coli*. These nanoparticles were able to stop the multidrug-resistant strains of *E. coli* and *Pseudomonas aeruginosa* from spreading.^82,83^ An *E. coli*-free transcription/translation system demonstrated that Au–DAPT inhibited protein synthesis. The modes of action of Au-DAPT were investigated using gel electrophoresis, which demonstrated the potential of nanoparticles to attach bacterial DNA; electron microscope images exhibiting nucleic acid leakage and Au-DAPT adhesion to chromosomes and ribosomes; and colorimetric analyses indicated Mg^2+^ selectivity during chelation, with the consequent membrane instability.^84^

Similarly, polymer-coated silver nanoparticles, also known as AgNPs, could destroy *E. coli* cells by blocking the citric acid cycle or the tricarboxylic acid cycle and the metabolism of amino acids. The surface of the AgNPs was modified using polymers to enhance their interactions with bacterial cells.^85,86^ The gene expression involved in the citric acid cycle and amino acid metabolism were suppressed, validating the action method and leading to cell death.

### 4.3 Reactive Oxygen Species (ROS) Production

**Figure 2:**
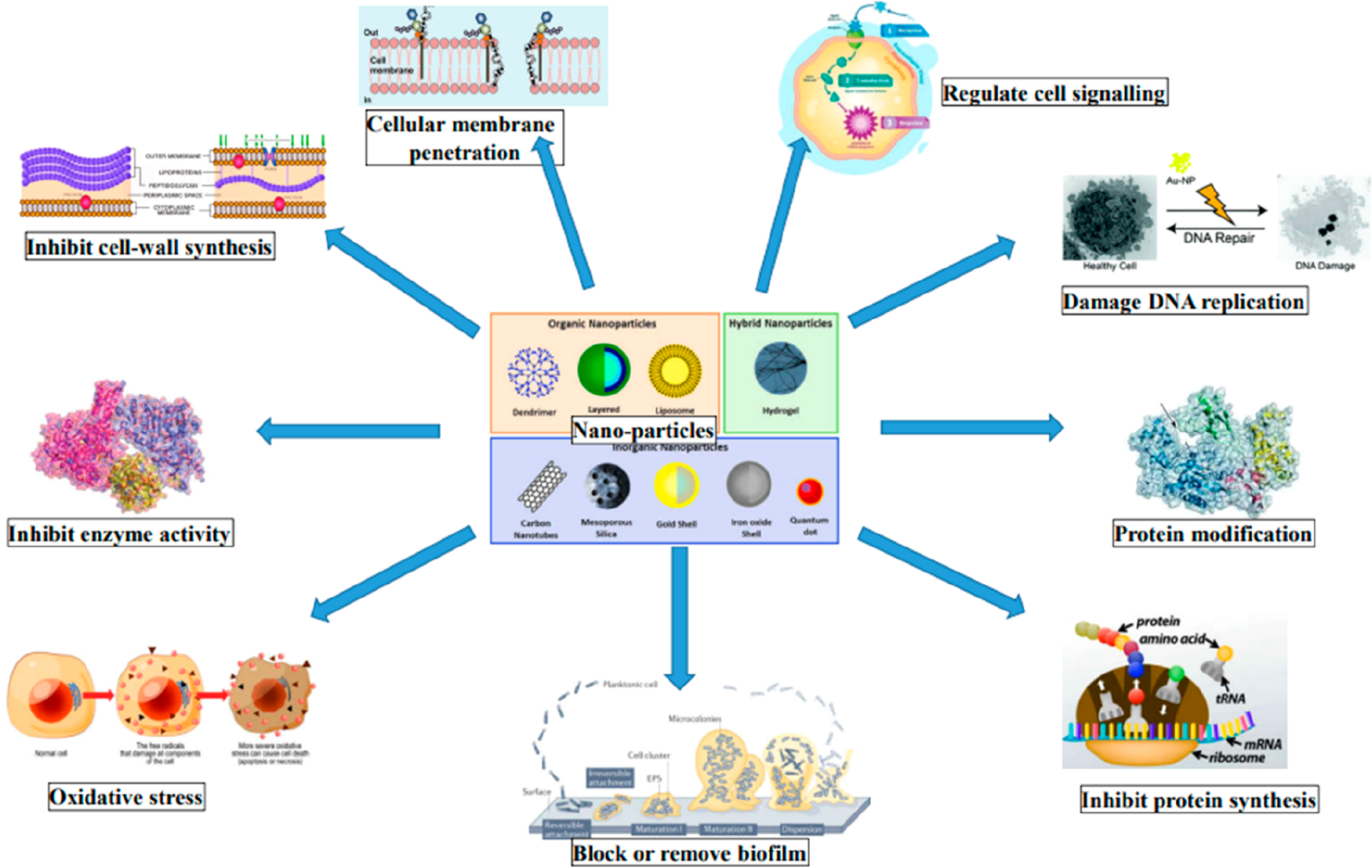
Different approaches used for antibacterial activity by nanoparticles.

ROS are oxidative metabolic byproducts that occur within cells. These byproducts affect cells’ differentiation, signaling, survival, and death.^87^ The buildup of toxic levels of reactive oxygen species (ROS) causes oxidative stress (Figure 2). ROS are capable of causing damage to cells through various processes, one of the most prominent of which is the interaction of protein thiols with hydroxyl radicals and superoxide, which deactivates membrane receptors. Nanoparticles can produce ROS through various methods, like intracellular organelles interactions, biomolecule oxidations using NADPH oxidase, and direct ROS production.^87−89^ Due to the inherent photocatalytic activity of certain metal-based nanoparticles, forming reactive oxygen species (ROS) is the primary antibacterial mechanism used by these nanoparticles (photodynamic therapy). An example of ROS-based antibacterial action is releasing unbound Cu^+^ ions from CuI nanoparticles.^90^ This process generates ROS and causes damage to *B. subtilis* and *E. coli* DNA and internal proteins. Antibacterial activity was also demonstrated by silver–zinc oxide nanocomposites against antibiotic-resistant *E. coli* and *S. aureus*. This action was attributed to powerful ROS production and the release of silver (Ag^+^) and zinc (Zn^2+^) ions by the nanocomposites.^91,92^ The combination of these processes then produced a chain reaction that resulted in bactericidal consequences, such as DNA replication inhibition, the disruption of protein function, the leakage of cellular biomolecules, and the destruction of cell membranes (Figure 2). Silver boosts ROS generation in bacteria by disrupting cellular donor ligands interacting with iron, like cysteine, and inducing the ejection of iron from [4Fe–4S] clusters and ROS synthesis. This iron release, in turn, causes an increase in ROS formation.^93^ Mesoporous silica can support and enhance gold nanoparticles’ catalytic activity and stability (AuNPs). The exterior of bifunctionalized mesoporous silica nanoparticles (MSNs) coated with AuNPs has been demonstrated to exhibit peroxidase- and oxidase-like properties, simultaneously eliminating Gram-negative and Gram-positive bacteria.^94,95^ This dual enzyme-like activity enhances the effectiveness of ROS synthesis and causes oxidative stress in bacteria.

### 4.4 Cell Wall and Membrane Disruption

The outer membrane of a bacterial cell has evolved to prevent the penetration of antimicrobial drugs. Gram-positive bacteria cell walls and Gram-negative bacteria cells have teichoic acids and LPS, respectively, which have phosphate groups and result in negatively charged bacterial surfaces.^96^ Because of this highly polar environment, hydrophobic antimicrobials have difficulty penetrating membranes, reducing their effectiveness against bacteria. Compared to mammalian cells, the bacterial cell surface is more negatively charged, making it easier for bacteria to engage in preferential electrostatic interactions with positively charged materials. Charge densities and hydrophobicity are critical in producing bacterial membrane-disrupting nanomaterials.^97−99^ Nanomaterials with high cationic surfaces and nanomaterials with excessively hydrophobic surfaces can bind to mammalian cells’ surfaces, decreasing selectivity. Cationic nanomaterials with strong amphiphilic levels have the potential to produce potent antibacterial activities while also exhibiting low levels of hemolysis and cytotoxicity.^100^ Targeting the planktonic bacteria with a negatively charged surface is the primary focus of many tactics based on nanomaterials. In one study, cationic and amphiphilic polycarbonates that are biodegradable and can self-assemble into cationic micellar nanoparticles were produced. These nanoparticles effectively treat methicillin-resistant *S. aureus* (MRSA).^101^ Electrostatic interactions between bacteria and these polymeric nanoparticles lead to the breakdown of the membranes and subsequent lysis of the cells (Figure 2). “Nano-knifes,” which are materials with sharp-pointed edges, are particularly effective in compromising the integrity of the membranes surrounding bacteria. It was reported that *Ralstonia solanacearum* cellular membrane could be disrupted using graphene oxide and carbon nanotubes (single-walled). This caused cytoplasmic leakage, ultimately leading to the bacteria’s death.^102^ It is expected that bacteria will have a limited capacity to become resistant to therapies that cause damage to the cell membrane. As a result, these techniques hold promise for usage over the long-term with a reduced likelihood of the development of bacterial resistance.

**Figure 3:**
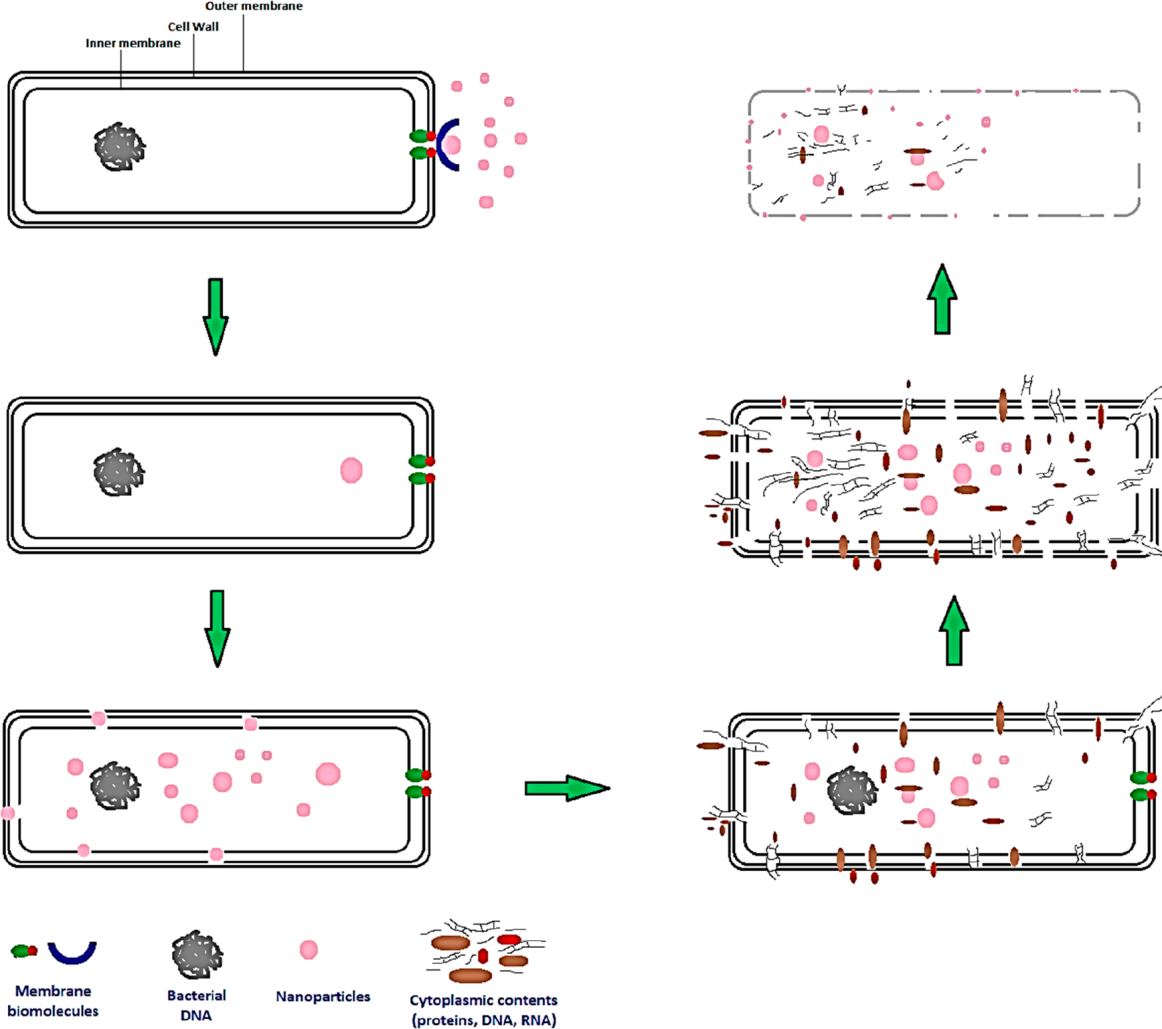
Pictorial presentation of nanoparticles’ cytotoxity effect on bacterial cell membrane and cell wall.

It has previously^103^ been shown that metal nanoparticles can physically interact with the cell membrane or the cell wall, as well as with intracellular components, as shown in Figure 3. Destruction of the cell wall is lethal to bacterial cells since it serves as a critical barrier between the cytoplasm and the outer environment, and it harbors essential metabolic activities like the electron transport chain and the regulated movement of molecules to and from the outside.^104,105^ There is a preference for electrostatic interactions between positively charged NPs and the negatively charged cell wall of Gram-positive and Gram-negative bacteria. The bacterial membrane is disrupted whenever it interacts with NPs because the particles are absorbed and enter the cell.^106^ Adsorption of NPs results in depolarization of the cell wall, which modifies the wall’s negative charge and makes it more permeable.

Consequently, the cell wall is broken down, and reactive oxygen species are formed. AgNPs have been demonstrated to stick to the cell wall, causing the cell wall to degrade and increasing the rate at which ions move through the cell membrane and into the cytosol.^31,107,108^ Ninganagouda et al.^109^ showed that AgNPs could attach themselves to the surfaces of bacteria (*E. coli*), leading to the death of the bacteria by the rupture of their cell membranes and the release of their internal components. Other studies^110,111^ have established that MgONPs and Mg(OH)_2_NPs can trigger cell death through electrostatic adsorption onto the cell wall rather than entering it. Another process related to physical contact is the cellular absorption of NPs, which occurs when NPs are small enough to pass through the cell membrane. Mukha et al.^112^ demonstrated that the antibacterial action of AgNPs with a size less than 10 nm is caused by membrane disruption and the AgNPs’ ability to penetrate the cell.

Similarly, Dong et al.^113^ examined NPs of varying sizes and found that smaller AgNPs were more potent because they could penetrate the cell membrane. Oves et al.^114^ synthesized silver nanoparticles (AgNPs) with a spherical form, a size of around 35 nm, and bacterial exopolysaccharides. They revealed that the generation of ROS within the bacterial cells is the cause of the antibacterial action of these NPs against *B. subtilis* and methicillin-resistant *Staphylococcus aureus* (MRSA). In addition, they demonstrated that NPs have good characteristics against the development of biofilms. In separate research,^115^ it was shown that the bactericidal activity of gold nanoparticles (Au NPs) against *E. coli* was caused by the suppression of ribosome subunits, in addition to the change of membrane and ATPase activities.

### 4.5 Delivery of Therapeutic Agents

The Food and Drug Administration (FDA) has approved several therapeutics that incorporate nanotechnology as “nanodrugs.” These “nanodrugs,” particularly liposomal nanoformulations, have been used to treat different diseases, including cancer.^116^ Along the same lines, nanoparticles have the potential to act as carriers for the delivery of antimicrobial agents. Nanomaterials can either contain therapeutics within their structures or bind them to their surfaces. These agents are protected from enzymes and chemicals that could otherwise break them down by the presence of nanomaterials.^117^ Such a response can improve a drug’s therapeutic efficiency, allowing a reduced dose to achieve the equivalent therapeutic effect while minimizing the host’s toxicity. Antibiotics that generally present several pharmacological challenges can improve their stability, solubility, and biocompatibility through delivery methods. Nanocarriers can reduce drug resistance by delivering therapies with diverse modes of action and by minimizing sub-inhibitory drug exposure to bacteria.^118,119^ Nanoparticles of poly(lactide-coglycolide) or (PLGA) loaded with gentamicin showed enhanced antibacterial efficacy against *P. aeruginosa* in both in vitro and in vivo studies.^120^

Consequently, levofloxacin packed inside silver core-embedded MSNs (Ag@MSNs@LEVO) effectively treated MDR *E. coli* isolates; the mixture had a synergistic antibacterial effect. Silver acted as a carrier and produced antibacterial silver ions. Treatment with Ag@MSNs@LEVO decreased bacterial load by three times, decreased spleen and peritoneum damage, and showed minimal toxicity in an in vivo mouse peritonitis model.^121^ Similarly, ampicillin was immobilized on the surface of AuNPs and AgNPs to develop broad antibacterial medicines that bypass the resistance strategies of methicillin-resistant *Staphylococcus aureus*, *Pseudomonas aeruginosa*, and *Enterobacter aerogenes*.^122^ Medical specificity and delivery efficacy can be improved by releasing medicine in response to specific stimulation. The infection sites of bacteria are slightly acidic and can be attacked by antibacterial agents. Vancomycin was enclosed in PLGA–PLH–PEG triblock copolymer, which is a pH-responsive polymer (poly(d,llactic-*co*-glycolic acid)-*b*-poly(l-histidine)-*b*-polyethylene glycol). The release of vancomycin was dependent upon association with the acidic infection area, which served as a target for administering vancomycin.^123^ PLGA was selected due to its minimal toxic effects and ease of surface modification. PEG minimized off-target contacts, extending circulatory duration. In a weakly acidic environment, specific protonation of PLH’s imidazole groups produced a stimuli-responsive response. In addition to charge-switching functionality, biomaterials like chitosan nanoparticles can discharge vancomycin in response to changes in pH.^123,124^ Additionally, bacterial toxins have the ability to act as a signal for the secretion of antibiotic molecules. DSPE-PEG3400 and lecithin utilized for fatty acid capping, generating liposome-based nanoreactors that emit rifampin and CaO_2_ in the vicinity of *S. aureus* produced toxin. This technique targeted harmful bacteria, as shown by its antibiotic effectiveness on MRSA and limited impact on *B. subtilis*, nonpathogenic strain.^125^ Nanomaterials offer several bactericidal methods to fight microorganisms and circumvent antimicrobial resistance. Novel antimicrobial agents can be designed in a variety of ways by manipulating their size, shape, and surface qualities.

## 5 Combating Planktonic Bacteria

Infections acquired in hospitals and resistance to medication provide a complex treatment challenge. Most nosocomial infections are caused by a group of pathogens known collectively as “ESKAPE pathogens.” These pathogens include *Enterococcus faecium*, *Staphylococcus aureus*, *Klebsiella pneumoniae*, *Acinetobacter baumannii*, *Pseudomonas aeruginosa*, and *Enterobacter* species.^126^ The high incidence of antibiotic resistance development, particularly for drugs regarded as the last choice, limits the treatment choices for infections caused by these organisms; further, it worsens the condition of usually immunocompromised patients. Numerous research has been conducted to investigate the effectiveness of nanomaterials in combating ESKAPE infections.^108^ In this aspect, nanomaterials have the potential to offer a savior for therapeutic design, as there is seen to be very little or no resistance development when using techniques that are based on nanomaterials.^6,12,18^

In one study, structurally nanoengineered antimicrobial peptide polymers (SNAPPs) were shown to be effective against ESKAPE infections (*Enterococcus faecium*, *Staphylococcus aureus*, *Klebsiella pneumoniae*, *Acinetobacter baumannii*, *Pseudomonas aeruginosa*, and *Enterobacter* spp.), a group of MDR Gram-negative bacteria both in vitro and in an in vivo peritonitis model of the mouse.^4^ Researchers have artificially synthesized antimicrobial peptide-derived nanoparticles. These lysine and valine-based nanoparticles can be self-assembled into unimolecular, star-shaped structures. This allows them to mimic the properties of antimicrobial peptides. Some of the bactericidal actions that SNAPPs are believed to trigger include apoptotic-like cell death, influx regulation or ion-efflux disturbance, and outer and inner cellular membrane disruption.^4^ The multimodal antibacterial action creates a significant barrier to resistance that SNAPPs are hypothesized to possess. Liposome-based nanoparticles are another potential technology that, by effective drug administration, can restore the potency of antibiotics such as ceftazidime, imipenem, and cefepime against multidrug-resistant *P. aeruginosa*, amikacin for *K. pneumoniae*, and chloramphenicol for MRSA-65.^12^ A practical method of treating a *P. aeruginosa* infection of the lungs in an in vivo mouse model was found to be the delivery of antimicrobial peptides through the use of PLGA nanoparticles.^127^

## 6 Combating Intracellular Bacteria

Systemic infections can be caused by bacteria living inside mammalian cells. *Salmonella enterica* (serovar: Typhimurium) is a prevalent example of a facultative intracellular pathogen. Each year, it is responsible for infecting millions of people worldwide with potentially fatal food-borne illnesses.^128,129^*Salmonella* species, including macrophages, can survive and replicate within their hosts’ cells. Intracellular bacteria complicate medication because several antibiotics cannot permeate mammalian cell membranes and are actively exiled from the host cell, making intracellular bacteria treatment more difficult. Nanomaterials offer a potential solution to this problem because of their excellent drug-loading capacity and ability to infiltrate eukaryotic cells. Docosanoic acid solid lipid nanoparticles loaded with enrofloxacin were used as an example of a nanomaterial-based treatment for intracellular infections.^130^ These nanoparticles improved enrofloxacin’s efficiency to enter cells by a magnitude of 40, leading to more effective *Salmonella* destruction in macrophages. In another method, the colistin antibiotic is placed inside liposomes that were functionalized with a protein derived from bacteria.^131^ It encouraged colistin’s internalization into eukaryotic cells, resulting in therapeutic production with high oral bioavailability.

Another method involved encasing gentamicin-loaded MSN in lipid bilayers that are responsive to bacterial toxins. Another example of an intracellular pathogen that can persist within its host’s macrophages is *Mycobacterium tuberculosis*, which is responsible for tuberculosis transmission.^132^ Nanomaterials are active against intracellular *Mycobacterium* species in several research studies. In accordance with the findings of one investigation, a library of cationic polycarbonate nanostructures in the shape of stars possessed both broad-spectrum antibacterial action and low hemolysis rates.^133^ In another work, AgNPs and zinc oxide nanoparticles encased in PLGA were employed to transport the antituberculosis rifampin into *M. tuberculosis*-infected alveolar macrophages.^133,134^ The antibacterial effects were enhanced by the potential of silver nanoparticles and zinc oxide nanoparticles to engage with and weaken the integrity of bacterial membranes. Nanomaterial-based techniques to fight additional intracellular infections have also been explored. For example, in in vivo mouse infection models, peptide-loaded AuNP-DNA aptamer antimicrobial conjugates displayed usefulness toward intracellular *Salmonella* species and *Vibrio vulnificus*.^135,136^ Another example demonstrates that intracellular *Listeria* monocytogenes and *P. aeruginosa* can be eliminated using gentamicin-loaded AuNPs that have been coated with phosphatidylcholine.^37^

## 7 Strategies Used to Treat Biofilms

Infections caused by MDR biofilms provide a challenging problem for therapeutic intervention. The extracellular polymeric substances (EPS) are made up of biopolymers such as nucleic acids, proteins, and polysaccharides, and they function as a three-dimensional protective scaffold for bacteria. The matrix made of EPS may act as a defense mechanism against some cellular and small-molecule attacks (like antibiotics, for instance). Bacteria incorporated into the matrix can engage in synergistic interactions, communicate with one another at the cell level, and pass on resistance genes.^12,46,78^ Several fundamental parameters, including size and electrostatic interactions, significantly influence nanomaterials’ biofilm penetration profiles. The matrix contains many negatively charged components and hydrophobic groups, and it also has many pores filled with water to make it easier for nutrients to move around. In addition, the deeper layers of the matrix have a lower supply of oxygen and nutrients, which induces the creation of dormant persister cells.^78−81^ These cells promote antimicrobial tolerance and resistance. In order to remove biofilms, it is necessary to find a way to circumvent the physical barrier that they present. Changing the functioning of the surface of nanoparticles or designing them differently can make biofilm penetration easier. In general, uncharged nanoparticles smaller than 350 nm and cationic nanoparticles have superior mobility across the pores in biofilms.^12^

### 7.1 Targeting Resident Pathogens

Nanomaterials have the potential to combine with bacteria and impose the healing processes previously mentioned for planktonic bacteria when they penetrate biofilms. For example, nanoparticles of poly(oxanorborneneimide) could eliminate MDR biofilms of the methicillin-resistant *S. aureus*, *E. cloacae*, and *P. aeruginosa* complex due to their effective biofilm penetrating characteristic and bacterial membrane-damaging ability.^100^ In an alternative technique, stimuli-responsive nanoparticles were used to activate bactericidal activities in a time- and space-controlled fashion. A pH-responsive silver nanoantibiotic was produced by employing self-assembly silver-nanoclusters in conjunction with the switchable charged-ligand PEG-poly(amino-propyl imidazole-aspartate)-polyalanine.^137^ The hydronation of imidazole groups of biofilms in the low-pH microenvironment caused the breakdown of pH-responsive silver nanoantibiotics. It happened due to electrostatic repulsion with silver ions. The dissociation of the silver nanoclusters into small units allowed for biofilm penetration, which resulted in the death of firmly embedded MRSA bacteria.^138^

Similarly, the introduction of an externally applied magnetic field made it easier for AgNPs to penetrate biofilms. The silver conferred an antibacterial effect on the nanoparticles. It is also possible for nanomaterials to supply medicines to bacterial cells that are implanted within an EPS matrix. Carvacrol oil, oregano essential oil, and thyme essential oil are three examples of potent antimicrobial oils that are unable to or poorly penetrate biofilms.^87^ It was reported that multiple drug-resistant biofilms were successfully eradicated utilizing carvacrol in oil-in-water cross-linked polymeric nanocomposites with limited damage to mammalian cells for Gram-positive and Gram-negative bacteria infections.^138^ Guanidinium contributed to the cationic nanocomposite. Maleimide groups cross-linked and biodegraded the nanocomposite, while tetra-ethylene-glycol-monomethyl ether made it hydrophilic. The polymer improved Carvacrol oil’s solubility, stability, biodegradability, and antibacterial effectiveness, which also helped it penetrate the biofilm.^139^

### 7.2 EPS Matrix Disruption

EPS matrix disruption is another treatment option for biofilms that can be used in addition to eliminating the bacteria there. After treatment, the residual EPS scaffold can be inhabited and colonized by various types of bacteria.^18^ Several nanomaterial-based methods, such as matrix-degrading enzymes and mechanical disruption, can be used to disperse the EPS matrix. These matrix-degrading enzymes involved protease, hydrolase, and DNase. In order to specifically target *P. aeruginosa* biofilms, PLGA nanoparticles contain ciprofloxacin and are functionalized with DNase I developed.^140^ Extracellular DNA was damaged by DNase I, which made the three-dimensional network weak and open to attack by ciprofloxacin.

Similarly, *Pseudomonas fluorescens* biofilms were broken up by AuNPs that had been functionalized with proteinase K.^141^ Alternately, the application of DC and AC magnetic fields produced nanoparticles (magnetic iron-oxide), resulting in the destruction of MRSA biofilms.^142^ The “shield breakers” were magnetic iron oxide nanoparticles traveling over the 3D network. These nanoparticles destroyed biofilms through the process of static friction. The introduction of magnetic iron-oxide nanoparticles triggered a localized rise in temperature into an AC magnetic field. This led to the release of cells that were embedded within the particles. Because these magnetic iron oxide nanoparticles do not kill bacteria as part of their mechanisms of action, the system provides an antibiofilm method that can be used over the long-term and may circumvent the development of resistance.^143^ Interrupting bacterial communication networks is a viable technique for combating the creation of biofilms because these systems are crucial for coordinated bacterial actions, such as colonization and the development of biofilms. Bacteria interact with one another through a mechanism known as quorum sensing. This process can be disrupted to stop biofilms’ production or make them disperse.^143^ In accordance with the findings of one investigation, inhibiting quorum sensing can effectively mute bacterial transmission. The communication between *Vibrio fischeri* cells was inhibited by silicon dioxide nanoparticles coated with beta-cyclodextrin.^144^ Bioluminescence is exhibited by *V. fischeri* and is determined by the population density of the organism. This bioluminescence can be observed due to acyl-homoserine lactone working as a signaling molecule during quorum sensing. The action of acyl-homoserine lactone is inhibited because a group of beta-cyclodextrin connected to silicon dioxide nanoparticles binds to the molecule.

Consequently, the amount of light given off by *V. fischeri* was diminished. In addition, there was a reduction in the activity of the luminescence genes luxA and luxR. Nanomaterials, which have a wide range of controllable properties, offer a versatile toolkit for combating various biofilm diseases. It was demonstrated that suppressing cell-signaling molecules using chitosan-, metal-, or liposome-based nanoparticles prevents biofilms and virulence factors. Nanomaterial penetration characteristics are a good indicator of whether biofilm removal will succeed. Nanoparticle dispersion throughout the biofilm is primarily influenced by size and amphiphilicity. Nanoparticle interactions with EPS rely on biofilm type, which varies with bacterial species and strain.^97−99^

## 8 Biofilm Infection Control

Biofilms are a significant factor in the development of chronic and long-lasting infections. The infections number are linked to biofilms keeps rising from one year to the next. Bacteria can develop biofilms on and within human tissues and organs, such as respiratory tract linings, digestive tract linings, oral cavities, and skin surfaces.^8,24,97^ Nanotherapeutic techniques have emerged as a viable therapy for biofilm infections in light of our increased knowledge of medical biofilms.^145^

### 8.1 Oral Biofilms

The oral cavity is one of the most common sites for the development of biofilms, and *Streptococcus mutans* is a prevalent example of a pathogen that can be found in oral biofilms. The deterioration of tooth enamel, which ultimately leads to dental caries, is caused by the acidic microenvironment present in dental biofilms, also known as plaque.^146^ Using the highly acidic environment of oral biofilms has allowed for developing nanoparticle-based techniques for treating infections associated with oral biofilms. The antibiotic doxycycline was delivered to oral biofilms of *P. gingivalis* using liposomes coated with quaternary ammonium-modified chitosan.^147,148^ Chitosan’s residual amines contribute to pH-responsive groups, which become protonated when exposed to an acidic environment. This results in an activity that is pH-dependent. These nanocarriers have been used for dental caries treatment by transporting chlorhexidine and farnesol.^149^ They are made from pH-sensitive block copolymers that firmly attach to hydroxyapatite, a negatively charged material. Studies on oral biofilm treatment also investigate nanoparticles that have the potential to trigger ROS generation and EPS matrix disintegration. For example, the use of catalytic nanoparticles composed of biocompatible Fe_3_O_4_ was taken to use in order to catalyze the in situ generations of free radicals from H_2_O_2_. This resulted in a drop in the number of *S. mutans* biofilms that were present.^150^ Iron oxide nanoparticles’ functionalization with oral soft tissues and stability in aqueous formulations were strengthened by coating them with dextran.^144^ Based on iron and licensed by the FDA, this nanoparticle has a pH-dependent peroxidase-like feature, enabling it to give localized catalytic activity. This study showed that ferumoxytol could permeate through biofilm matrices and create free radicals from H_2_O_2_, leading to the death of bacteria in their natural environment and the destruction of EPS.^151^ A human-derived in vivo and in vitro mice dental cavity/caries model demonstrated effectiveness in reducing acid erosion to the enamel and suppressing tooth decay without affecting the oral microbiome, as well as safety to periodontal and mucosa tissues.^152^

### 8.2 Wound Biofilms

Wound infections afflict over 300 million individuals worldwide, and it is anticipated that treating these infections will cost at least $25 billion just in the United States of America.^153^ Necrotic tissue helps in the adhesion of bacteria in these infections while also providing nutrients that delay the healing of wounds by preventing re-epithelialization and extending inflammation. The treatment of wound infections utilizes AgNPs that have been integrated into hydrogels or wound dressings.^154^ Additional types of nanoparticles have also been the subject of increasing research for treating wounds affected by biofilm. For instance, *P. aeruginosa* and *S. aureus* biofilms could not form when copper particles were inserted into biodegradable nanofibers, and any biofilms that had already grown were destroyed by the copper particles.^155^ In order to demonstrate that this method may be used in wound dressings, more in vitro and in vivo investigations are currently being conducted. In another method, the amphiphilic nanoparticle DA95B5 is used to remove MRSA biofilms that have already formed.^156^ This is achieved through a process called “nanoscale bacterial debridement.” When DA95B5 diffuses through the EPS, it weakens the adhesion of bacteria to the matrix, dispersing the biofilm. Dispersal of MRSA biofilms was shown to be successful in an in vivo mouse-excisional-wound biofilm-model. Hydrogel pad dressings soaked in DA95B5 significantly decreased the number of bacteria up to four logs present in mice. Nanoparticles showed very little lysis of eukaryotic cells in vitro and had a very low level of toxicity in animals. In order to further nanoparticle-based treatment strategies for wound infections, it may be beneficial to combine these nanoparticles with molecules (such as extracellular matrix mimics, anti-inflammatory molecules, and growth factors) that speed up the wound healing process.

An example of this would be incorporating a pH-responsive antimicrobial nanofiber network into a hydrogel that was then loaded with cypate and proline.^157^ The octapeptide IKFQFHFD is self-assembled to form this network. The octapeptide has an inherent antimicrobial property that works by disrupting cell walls and membranes; cypate is a photothermal drug responsible for EPS-matrix disruption.^157^ This was presented in a diabetic mouse model in an in vivo environment, where MRSA biofilms were treated with hydrogel, accelerating the chronic wound healing process.

## 9 Toward Clinical Translation (Advantages and Disadvantages of NPs in Antimicrobial therapy)

Exploration of antimicrobial nanomaterials as potential treatments for multidrug-resistant (MDR) planktonic bacteria and biofilm infections has recently seen a significant uptick (Table 3). Providing clinical feasibility for using nanoparticles requires the development of suitable in vivo and in vitro models that represent the safety and efficacy of nanoparticles.^77^ Most research has been carried out in vitro with animal models and human trials. Several reviews have provided in vitro and in vivo models to investigate, and these models differ depending on the targeted infection type.^12,120,158^ Nanocarriers for antibiotic delivery or antimicrobial AgNPs comprise most formulations currently undergoing clinical testing. Although, NPs have the potential to cure bacterial infections, but numerous hurdles remain for their effective translation to the clinic, including additional examination of NP interactions with cells, tissues, and organs; optimal dosage; acceptable delivery routes; and toxicity after acute and long-term exposure.^159,160^ Nanoparticles (NPs) have shown potential in antimicrobial therapy, but their use also has some disadvantages (Table 3). Here are some of them. Toxicity: Some types of NPs can be toxic to cells, leading to undesirable side effects. The toxicity of NPs can be influenced by size, shape, and surface charge.^159^ Potential for resistance: There is a concern that bacteria may develop resistance to NPs, just as they do with antibiotics. This could potentially limit the long-term effectiveness of NP-based antimicrobial therapies.^159,161^ Limited efficacy against some bacteria: NPs may be less effective against certain types of bacteria or bacterial biofilms, making treatment more challenging.^117^ Difficulty in targeting specific cells: It can be challenging to target NPs to specific cells or tissues in the body, limiting their effectiveness and increasing the risk of side effects. Cost: The development and production of NPs can be expensive, limiting their accessibility and affordability for some patients. Regulatory challenges: The use of NPs in antimicrobial therapy is a relatively new area of research, and regulatory approval for their use may be challenging. This can limit their availability and use in clinical settings.^162^

**Table 3:** Beneficial and Adverse Effects of Nanoparticles in Therapeutic Usage^7,15,33,37,45,55,57,58,62,88,158^

beneficial effects	adverse effects
low immunosuppression	insufficient availability of characterization methods that are not influenced by the characteristics of nanoparticles
controlled drug release	
better solubility	
a wide range of therapeutic effectiveness	nanotoxicity to organs and metabolic processes
longer lifespan of therapeutic effects as a result of the slow elimination	maximum therapeutic benefit from locally administered drugs through extensive systemic exposure
the capability of penetrating biological barriers (e.g., blood–brain barrier)	
reduced risk of developing bacterial resistance	deposition of nanomaterials administered intravenously in the body’s organs and cells
comparatively minimal adverse effects as contrasted to chemical antimicrobials	
delivery of medications to specific locations through the accumulation of those medications	

In contrast to traditional antibiotics, NPs have several significant benefits due to their unique physical structure. The current status of nanoparticles demonstrates a promising potential for use soon in the topical treatment of skin ailments.^163^ Several people have tried to use NPs on the fibers, fabrics, and electronics that come into direct touch with the human body.^164−166^ Nonetheless, the systemic delivery of NPs still requires consideration of several factors. For clinical translation, adequate regulations for the synthesis and scaled-up production of these nanomaterials, characterization of their physicochemical characteristics and their implications on biomaterials, validation of nanotoxicological tests, and methodologies to evaluate in vitro and in vivo findings are anticipated.^167^ Future preclinical research must include therapeutic effectiveness measures in clinical trials, as well as the safety of NP systems. Moreover, in terms of therapeutic effectiveness, it is essential to evaluate the financial ramifications of the clinical translation of these NPs.^168−170^ It is also essential to research the negative consequences of nanoparticles that may be responsible for the propagation of MDR, which may result in additional threats to both public health and the environment (Table 3).

## 10 Conclusions and Future Perspective

Nanomaterials offer a promising alternative approach to combat persistent multiple drug-resistant bacteria and biofilm infections resistant to traditional antibiotics. Nanoparticles can be designed with specific surface functions to enhance the therapeutic impact and minimize toxicity to the host. The multimodal antibacterial mechanisms of nanomaterials can significantly slow down or halt the development of drug resistance, making them a potential solution to the challenges of the postantibiotic era. However, the clinical use of nanomaterials still faces obstacles, including a lack of understanding concerning nanoparticle toxicity, clearance, and metabolism. In addition, an in-depth understanding of the pharmacokinetics and pharmacodynamics of nanoparticles is necessary to translate this knowledge into clinical practice. Stimuli-responsive nanoparticles that capitalize on the distinct microenvironments at infection sites offer a potential solution to target multidrug-resistant bacteria.

Collaboration between chemists, biomedical researchers, microbiologists, and engineers is crucial to develop effective antimicrobial nanomaterials. Similarly, the collaboration between basic, translational, and industrial research institutions is essential to bring antimicrobial nanomaterials to clinical use. In conclusion, nanomaterial-based treatments offer a viable alternative to antibiotics for severe diseases, and the development of antimicrobial nanomaterials has the potential to revolutionize the medical field. Despite the obstacles that still exist, the potential benefits of using nanomaterials to combat antibiotic resistance cannot be ignored, and further research in this area is essential to overcome the challenges and bring these therapies to clinical use.

## References

[ref1] Bankier C.; Matharu R. K.; Cheong Y. K.; Ren G. G.; Cloutman-Green E.; Ciric L. Synergistic Antibacterial Effects of Metallic Nanoparticle Combinations. Sci. Rep. 2019, 9 (1), 16074 10.1038/s41598-019-52473-2. 31690845 PMC6831564

[ref2] Christensen S. B. Drugs That Changed Society: History and Current Status of the Early Antibiotics: Salvarsan, Sulfonamides, and beta-Lactams. Molecules. 2021, 26 (19), 6057 10.3390/molecules26196057. 34641601 PMC8512414

[ref3] Rutten A.; Kirchner T.; Musiol-Kroll E. M. Overview on Strategies and Assays for Antibiotic Discovery. Pharmaceuticals (Basel). 2022, 15 (10), 1302 10.3390/ph15101302. 36297414 PMC9607151

[ref4] Lam S. J.; O’Brien-Simpson N. M.; Pantarat N.; Sulistio A.; Wong E. H. H.; Chen Y. Y.; Lenzo J. C.; Holden J. A.; Blencowe A.; Reynolds E. C.; Qiao G. G. Combating multidrug-resistant Gram-negative bacteria with structurally nanoengineered antimicrobial peptide polymers. Nature. Microbiology 2016, 1, 16162 10.1038/nmicrobiol.2016.162. 27617798

[ref5] Pushparaj Selvadoss P.; Nellore J.; Balaraman Ravindrran M.; Sekar U.; Tippabathani J. Enhancement of antimicrobial activity by liposomal oleic acid-loaded antibiotics for the treatment of multidrug-resistant *Pseudomonas aeruginosa*. Artificial Cells, Nanomedicine and Biotechnology. 2018, 46 (2), 268–73. 10.1080/21691401.2017.1307209. 28362119

[ref6] Naylor N. R.; Atun R.; Zhu N.; Kulasabanathan K.; Silva S.; Chatterjee A.; Knight G. M.; Robotham J. V. Estimating the burden of antimicrobial resistance: a systematic literature review. Antimicrobial resistance and infection control. 2018, 7, 58 10.1186/s13756-018-0336-y. 29713465 PMC5918775

[ref7] Makabenta J. M. V.; Nabawy A.; Li C. H.; Schmidt-Malan S.; Patel R.; Rotello V. M. Nanomaterial-based therapeutics for antibiotic-resistant bacterial infections. Nat. Rev. Microbiol. 2021, 19 (1), 23–36. 10.1038/s41579-020-0420-1. 32814862 PMC8559572

[ref8] Rout B.; Liu C. H.; Wu W. C. Photosensitizer in lipid nanoparticle: a nano-scaled approach to antibacterial function. Sci. Rep. 2017, 7 (1), 7892 10.1038/s41598-017-07444-w. 28801673 PMC5554217

[ref9] Michailidu J.; Matatkova O.; Kolouchova I.; Masak J.; Cejkova A. Silver Nanoparticle Production Mediated by Vitis vinifera Cane Extract: Characterization and Antibacterial Activity Evaluation. Plants (Basel) 2022, 11 (3), 443 10.3390/plants11030443. 35161424 PMC8840298

[ref10] Del Carpio-Perochena A.; Kishen A.; Shrestha A.; Bramante C. M. Antibacterial Properties Associated with Chitosan Nanoparticle Treatment on Root Dentin and 2 Types of Endodontic Sealers. J. Endod. 2015, 41 (8), 1353–8. 10.1016/j.joen.2015.03.020. 25958178

[ref11] Wang L. S.; Gupta A.; Rotello V. M. Nanomaterials for the Treatment of Bacterial Biofilms. ACS Infectious Diseases. 2016, 2 (1), 3–4. 10.1021/acsinfecdis.5b00116. 27622944 PMC5848070

[ref12] Qi M.; Chi M.; Sun X.; Xie X.; Weir M. D.; Oates T. W.; Zhou Y.; Wang L.; Bai Y.; Xu H. H. Novel nanomaterial-based antibacterial photodynamic therapies to combat oral bacterial biofilms and infectious diseases. Int. J. Nanomedicine. 2019, 14, 6937–56. 10.2147/IJN.S212807. 31695368 PMC6718167

[ref13] Mirzaie A.; Peirovi N.; Akbarzadeh I.; Moghtaderi M.; Heidari F.; Yeganeh F. E.; Noorbazargan H.; Mirzazadeh S.; Bakhtiari R. Preparation and optimization of ciprofloxacin encapsulated niosomes: A new approach for enhanced antibacterial activity, biofilm inhibition and reduced antibiotic resistance in ciprofloxacin-resistant methicillin-resistance *Staphylococcus aureus*. Bioorg Chem. 2020, 103, 104231 10.1016/j.bioorg.2020.104231. 32882442

[ref14] Patel R. Biofilms and antimicrobial resistance. Clinical Orthopaedics and Related Research. 2005, 437, 41 10.1097/01.blo.0000175714.68624.74. 16056024

[ref15] Van Acker H.; Van Dijck P.; Coenye T. Molecular mechanisms of antimicrobial tolerance and resistance in bacterial and fungal biofilms. Trends in Microbiology. 2014, 22 (6), 326–33. 10.1016/j.tim.2014.02.001. 24598086

[ref16] Cai J.; Huang H.; Song W.; Hu H.; Chen J.; Zhang L.; Li P.; Wu R.; Wu C. Preparation and evaluation of lipid polymer nanoparticles for eradicating H. pylori biofilm and impairing antibacterial resistance in vitro. Int. J. Pharm. 2015, 495 (2), 728–37. 10.1016/j.ijpharm.2015.09.055. 26417849

[ref17] Mohamed H. M. A.; Alnasser S. M.; Abd-Elhafeez H. H.; Alotaibi M.; Batiha G. E.; Younis W. Detection of beta-Lactamase Resistance and Biofilm Genes in *Pseudomonas Species* Isolated from Chickens. Microorganisms. 2022, 10 (10), 1975 10.3390/microorganisms10101975. 36296251 PMC9611058

[ref18] Fruh R.; Anderson A.; Cieplik F.; Hellwig E.; Wittmer A.; Vach K.; Al-Ahmad A. Antibiotic Resistance of Selected Bacteria after Treatment of the Supragingival Biofilm with Subinhibitory Chlorhexidine Concentrations. Antibiotics (Basel) 2022, 11 (10), 1420 10.3390/antibiotics11101420. 36290078 PMC9598507

[ref19] Sehmi S. K.; Noimark S.; Pike S. D.; Bear J. C.; Peveler W. J.; Williams C. K.; Shaffer M. S.; Allan E.; Parkin I. P.; MacRobert A. J. Enhancing the Antibacterial Activity of Light-Activated Surfaces Containing Crystal Violet and ZnO Nanoparticles: Investigation of Nanoparticle Size, Capping Ligand, and Dopants. ACS Omega. 2016, 1 (3), 334–43. 10.1021/acsomega.6b00017. 27840856 PMC5098237

[ref20] Karthikeyan C.; Varaprasad K.; Akbari-Fakhrabadi A.; Hameed A. S. H.; Sadiku R. Biomolecule chitosan, curcumin and ZnO-based antibacterial nanomaterial, via a one-pot process. Carbohydr. Polym. 2020, 249, 116825 10.1016/j.carbpol.2020.116825. 32933672

[ref21] Ventola C. L. The antibiotic resistance crisis: Part 1: causes and threats. P and T 2015, 40 (4), 277–83. 25859123 PMC4378521

[ref22] Lee V. C. The antibiotic resistance crisis: Part 2: Management strategies and new agents. P and T 2015, 40 (5), 344–352. 25987823 PMC4422635

[ref23] Bera S.; Mondal D. Stimuli-sensitive nanomaterials for antimicrobial drug delivery. Drug Targeting and Stimuli Sensitive Drug Delivery Systems 2018, 271–302. 10.1016/B978-0-12-813689-8.00007-0.

[ref24] Mabrouk M.; Rajendran R.; Soliman I. E.; Ashour M. M.; Beherei H. H.; Tohamy K. M.; Thomas S.; Kalarikkal N.; Arthanareeswaran G.; Das D. B. Nanoparticle- and Nanoporous-Membrane-Mediated Delivery of Therapeutics. Pharmaceutics. 2019, 11 (6), 294 10.3390/pharmaceutics11060294. 31234394 PMC6631283

[ref25] Xu D.; Wang T.; Wang S.; Jiang Y.; Wang Y.; Chen Y.; Bi Z.; Geng S. Antibacterial Effect of the Controlled Nanoscale Precipitates Obtained by Different Heat Treatment Schemes with a Ti-Based Nanomaterial, Ti-7.5Mo-5Cu Alloy. ACS Appl. Bio Mater. 2020, 3 (9), 6145–54. 10.1021/acsabm.0c00716. 35021747

[ref26] Pramanik A.; Laha D.; Bhattacharya D.; Pramanik P.; Karmakar P. A novel study of antibacterial activity of copper iodide nanoparticle mediated by DNA and membrane damage. Colloids and Surfaces B: Biointerfaces. 2012, 96, 50–5. 10.1016/j.colsurfb.2012.03.021. 22521682

[ref27] de Castro D. T.; Valente M. L.C.; Agnelli J. A. M.; Lovato da Silva C. H.; Watanabe E.; Siqueira R. L.; Alves O. L.; Holtz R. D.; dos Reis A. C. In vitro study of the antibacterial properties and impact strength of dental acrylic resins modified with a nanomaterial. J. Prosthet Dent. 2016, 115 (2), 238–46. 10.1016/j.prosdent.2015.09.003. 26545862

[ref28] Arasoglu T.; Derman S.; Mansuroglu B. Comparative evaluation of antibacterial activity of caffeic acid phenethyl ester and PLGA nanoparticle formulation by different methods. Nanotechnology. 2016, 27 (2), 025103 10.1088/0957-4484/27/2/025103. 26629915

[ref29] Radovic-Moreno A. F.; Lu T. K.; Puscasu V. A.; Yoon C. J.; Langer R.; Farokhzad O. C. Surface charge-switching polymeric nanoparticles for bacterial cell wall-targeted delivery of antibiotics. ACS Nano 2012, 6 (5), 4279–87. 10.1021/nn3008383. 22471841 PMC3779925

[ref30] Mitra S.; Mondal A. H.; Mukhopadhyay K. Mitigating the toxicity of palmitoylated analogue of alpha-melanocyte stimulating hormone(11–13) by conjugation with gold nanoparticle: characterisation and antibacterial efficacy against methicillin sensitive and resistant *Staphylococccus aureus*. World J. Microbiol. Biotechnol. 2022, 38 (11), 186 10.1007/s11274-022-03365-7. 35972627 PMC9379238

[ref31] Hasan N.; Cao J.; Lee J.; Hlaing S. P.; Oshi M. A.; Naeem M.; Ki M. H.; Lee B. L.; Jung Y.; Yoo J. W. Bacteria-Targeted Clindamycin Loaded Polymeric Nanoparticles: Effect of Surface Charge on Nanoparticle Adhesion to MRSA, Antibacterial Activity, and Wound Healing. Pharmaceutics. 2019, 11 (5), 236 10.3390/pharmaceutics11050236. 31096709 PMC6571677

[ref32] Hiwatashi N.; Kraja I.; Benedict P. A.; Dion G. R.; Bing R.; Rousseau B.; Amin M. R.; Nalband D. M.; Kirshenbaum K.; Branski R. C. Nanoparticle delivery of RNA-based therapeutics to alter the vocal fold tissue response to injury. Laryngoscope. 2018, 128 (5), E178–E183. 10.1002/lary.27047. 29238989 PMC5910268

[ref33] Saruchi; Kaur M.; Kumar V.; Ghfar A. A.; Pandey S. A Green Approach for the Synthesis of Silver Nanoparticle-Embedded Chitosan Bionanocomposite as a Potential Device for the Sustained Release of the Itraconazole Drug and Its Antibacterial Characteristics. Polymers (Basel). 2022, 14 (9), 1911 10.3390/polym14091911. 35567081 PMC9104402

[ref34] Tran D. T.; Jones I. P.; Preece J. A.; Johnston R. L.; Deplanche K.; Macaskie L. E. Configuration of microbially synthesized Pd-Au nanoparticles studied by STEM-based techniques. Nanotechnology. 2012, 23 (5), 055701 10.1088/0957-4484/23/5/055701. 22236722

[ref35] Teli M. D.; Sheikh J. Modified bamboo rayon-copper nanoparticle composites as antibacterial textiles. Int. J. Biol. Macromol. 2013, 61, 302–7. 10.1016/j.ijbiomac.2013.07.015. 23916646

[ref36] He H.; Tao G.; Wang Y.; Cai R.; Guo P.; Chen L.; Zuo H.; Zhao P.; Xia Q. In situ green synthesis and characterization of sericin-silver nanoparticle composite with effective antibacterial activity and good biocompatibility. Mater. Sci. Eng. C Mater. Biol. Appl. 2017, 80, 509–16. 10.1016/j.msec.2017.06.015. 28866194

[ref37] Lee B.; Park J.; Ryu M.; Kim S.; Joo M.; Yeom J. H.; Kim S.; Park Y.; Lee K.; Bae J. Antimicrobial peptide-loaded gold nanoparticle-DNA aptamer conjugates as highly effective antibacterial therapeutics against *Vibrio vulnificus*. Sci. Rep. 2017, 7 (1), 13572 10.1038/s41598-017-14127-z. 29051620 PMC5648795

[ref38] Bardhan S.; Pal K.; Roy S.; Das S.; Chakraborty A.; Karmakar P.; Basu R.; Das S. Nanoparticle Size-Dependent Antibacterial Activities in Natural Minerals. J. Nanosci Nanotechnol. 2019, 19 (11), 7112–22. 10.1166/jnn.2019.16658. 31039865

[ref39] Jayasinghe M. K.; Lee C. Y.; Tran T. T.; Tan R.; Chew S. M.; Yeo B. Z.; Loh W. X.; Pirisinu M.; Le M. T. The Role of in silico Research in Developing Nanoparticle-Based Therapeutics. Front Digit Health. 2022, 4, 838590 10.3389/fdgth.2022.838590. 35373184 PMC8965754

[ref40] Mao L.; Wang L.; Zhang M.; Ullah M. W.; Liu L.; Zhao W.; Li Y.; Ahmed A. A.; Cheng H.; Shi Z.; Yang G. In Situ Synthesized Selenium Nanoparticles-Decorated Bacterial Cellulose/Gelatin Hydrogel with Enhanced Antibacterial, Antioxidant, and Anti-Inflammatory Capabilities for Facilitating Skin Wound Healing. Adv. Healthc Mater. 2021, 10 (14), 2100402 10.1002/adhm.202100402. 34050616

[ref41] Yang K.; Han Q.; Chen B.; Zheng Y.; Zhang K.; Li Q.; Wang J. Antimicrobial hydrogels: promising materials for medical application. Int. J. Nanomedicine. 2018, 13, 2217–63. 10.2147/IJN.S154748. 29695904 PMC5905846

[ref42] Kasimanickam R. K.; Ranjan A.; Asokan G. V.; Kasimanickam V. R.; Kastelic J. P. Prevention and treatment of biofilms by hybrid- and nanotechnologies. Int. J. Nanomedicine. 2013, 8, 2809–19. 10.2147/IJN.S44100. 23946652 PMC3739460

[ref43] Aderibigbe B. A. Metal-Based Nanoparticles for the Treatment of Infectious Diseases. Molecules. 2017, 22 (8), 1370 10.3390/molecules22081370. 28820471 PMC6152252

[ref44] Yeh Y-C; Huang T-H; Yang S-C; Chen C-C; Fang J.-Y. Nano-Based Drug Delivery or Targeting to Eradicate Bacteria for Infection Mitigation: A Review of Recent Advances. Front. Chem. 2020, 8, 286 10.3389/fchem.2020.00286. 32391321 PMC7193053

[ref45] Meydan I.; Burhan H.; Gur T.; Seckin H.; Tanhaei B.; Sen F. Characterization of *Rheum ribes* with ZnO nanoparticle and its antidiabetic, antibacterial, DNA damage prevention and lipid peroxidation prevention activity of in vitro. Environ. Res. 2022, 204, 112363 10.1016/j.envres.2021.112363. 34774505

[ref46] Yamada R.; Nozaki K.; Horiuchi N.; Yamashita K.; Nemoto R.; Miura H.; Nagai A. Ag nanoparticle-coated zirconia for antibacterial prosthesis. Mater. Sci. Eng. C Mater. Biol. Appl. 2017, 78, 1054–60. 10.1016/j.msec.2017.04.149. 28575940

[ref47] Guo Z.; Chen Y.; Wang Y.; Jiang H.; Wang X. Advances and challenges in metallic nanomaterial synthesis and antibacterial applications. J. Mater. Chem. B 2020, 8 (22), 4764–77. 10.1039/D0TB00099J. 32207511

[ref48] Samanta A.; Podder S.; Kumarasamy M.; Ghosh C. K.; Lahiri D.; Roy P.; Bhattacharjee S.; Ghosh J.; Mukhopadhyay A. K. Au nanoparticle-decorated aragonite microdumbbells for enhanced antibacterial and anticancer activities. Mater. Sci. Eng. C Mater. Biol. Appl. 2019, 103, 109734 10.1016/j.msec.2019.05.019. 31349529

[ref49] Zhao H.; Zhang L.; Zheng S.; Chai S.; Wei J.; Zhong L.; He Y.; Xue J. Bacteriostatic activity and cytotoxicity of bacterial cellulose-chitosan film loaded with in-situ synthesized silver nanoparticles. Carbohydr. Polym. 2022, 281, 119017 10.1016/j.carbpol.2021.119017. 35074133

[ref50] Kumar V.; Jolivalt C.; Pulpytel J.; Jafari R.; Arefi-Khonsari F. Development of silver nanoparticle loaded antibacterial polymer mesh using plasma polymerization process. J. Biomed Mater. Res. A 2013, 101A (4), 1121–32. 10.1002/jbm.a.34419. 23015534

[ref51] Khan M. S.; Shah J. A.; Riaz N.; Butt T. A.; Khan A. J.; Khalifa W.; Gasmi H. H.; Latifee E. R.; Arshad M.; Al-Naghi A. A. A.; Ul-Hamid A.; Arshad M.; Bilal M. Synthesis and characterization of Fe-TiO2 nanomaterial: performance evaluation for RB5 decolorization and in vitro antibacterial studies. Nanomaterials 2021, 11 (2), 436 10.3390/nano11020436. 33572142 PMC7915575

[ref52] Peng C.; Pang R.; Li J.; Wang E. Current Advances on the Single-Atom Nanozyme and its Bio-Applications. Adv. Mater. [Online early access], 2023, e2211724, 10.1002/adma.202211724. 36773312

[ref53] Chen X.; Liao J.; Lin Y.; Zhang J.; Zheng C. Nanozyme’s catalytic activity at neutral pH: reaction substrates and application in sensing. Anal Bioanal Chem. [Online early access], 2023, 10.1007/s00216-023-04525-w. 36633622

[ref54] Kim H.; Lee E. H.; Lee S. W.; Deng Y. H.; Kwon H. B.; Lim Y. J.; Kong H.; Kim M. J. Antimicrobial efficacy of self-locomotive manganese oxide nanozyme-doped diatom microbubbler on orthodontic brackets in vitro. BMC Oral Health. 2023, 23 (1), 33 10.1186/s12903-023-02739-z. 36670429 PMC9854181

[ref55] Li Y.; Wang Q.; Qu X.; Zhang Q.; Zhang X. A metalloporphyrin and hydantoin functionalized nanozyme with synergistically enhanced bacterial inhibition. Biomater Sci. 2023, 11, 1785 10.1039/D2BM01337A. 36648752

[ref56] Zheng Y.; Liu W.; Qin Z.; Chen Y.; Jiang H.; Wang X. Mercaptopyrimidine-conjugated gold nanoclusters as nanoantibiotics for combating multidrug-resistant superbugs. Bioconjugate Chemistry. 2018, 29 (9), 3094–3103. 10.1021/acs.bioconjchem.8b00452. 30063328

[ref57] Zhang X.; Jiang X.; Croley T. R.; Boudreau M. D.; He W.; Cai J.; Li P.; Yin J. J. Ferroxidase-like and antibacterial activity of PtCu alloy nanoparticles. Journal of Environmental Science and Health, Part C 2019, 37 (2), 99–115. 10.1080/10590501.2019.1602991. 31099294

[ref58] Cai T.; Fang G.; Tian X.; Yin J.-J.; Chen C.; Ge C. Optimization of antibacterial efficacy of noble-metal-based core–shell nanostructures and effect of natural organic matter. ACS Nano 2019, 13 (11), 12694–12702. 10.1021/acsnano.9b04366. 31644267

[ref59] Qiu H.; Pu F.; Liu Z.; Liu X.; Dong K.; Liu C.; Ren J.; Qu X. Hydrogel-based artificial enzyme for combating bacteria and accelerating wound healing. Nano Research. 2020, 13, 496–502. 10.1007/s12274-020-2636-9.

[ref60] Han S. I.; Lee S. W.; Cho M. G.; Yoo J. M.; Oh M. H.; Jeong B.; Kim D.; Park O. K.; Kim J.; Namkoong E.; Jo J.; et al. Epitaxially strained CeO2/Mn3O4 nanocrystals as an enhanced antioxidant for radioprotection. Adv. Mater. 2020, 32 (31), 2001566 10.1002/adma.202001566. 32520432

[ref61] Luo Z.; Cui H.; Guo J.; Yao J.; Fang X.; Yan F.; Wang B.; Mao H. Poly (ionic liquid)/Ce-Based Antimicrobial Nanofibrous Membrane for Blocking Drug-Resistance Dissemination from MRSA-Infected Wounds. Advanced Functional Materials. 2021, 31 (23), 2100336 10.1002/adfm.202100336.

[ref62] Gao L.; Liu Y.; Kim D.; Li Y.; Hwang G.; Naha P. C.; Cormode D. P.; Koo H. Nanocatalysts promote *Streptococcus mutans* biofilm matrix degradation and enhance bacterial killing to suppress dental caries in vivo. Biomaterials. 2016, 101, 272–84. 10.1016/j.biomaterials.2016.05.051. 27294544 PMC4949957

[ref63] Xu Z.; Qiu Z.; Liu Q.; Huang Y.; Li D.; Shen X.; Fan K.; Xi J.; Gu Y.; Tang Y.; Jiang J.; et al. Converting organosulfur compounds to inorganic polysulfides against resistant bacterial infections. Nat. Commun. 2018, 9 (1), 3713 10.1038/s41467-018-06164-7. 30213949 PMC6137151

[ref64] Gao W.; He J.; Chen L.; Meng X.; Ma Y.; Cheng L.; Tu K.; Gao X.; Liu C.; Zhang M.; Fan K.; Pang D.-W.; Yan X. Deciphering the catalytic mechanism of superoxide dismutase activity of carbon dot nanozyme. Nat. Commun. 2023, 14 (1), 160 10.1038/s41467-023-35828-2. 36631476 PMC9834297

[ref65] Wang H.; Li P.; Yu D.; Zhang Y.; Wang Z.; Liu C.; Qiu H.; Liu Z.; Ren J.; Qu X. Unraveling the enzymatic activity of oxygenated carbon nanotubes and their application in the treatment of bacterial infections. Nano letters. 2018, 18 (6), 3344–51. 10.1021/acs.nanolett.7b05095. 29763562

[ref66] Wang L.; Gao F.; Wang A.; Chen X.; Li H.; Zhang X.; Zheng H.; Ji R.; Li B.; Yu X.; Liu J.; Gu Z.; Chen F.; Chen C. Defect-rich adhesive molybdenum disulfide/rGO vertical heterostructures with enhanced nanozyme activity for smart bacterial killing application. Adv. Mater. 2020, 32 (48), 2005423 10.1002/adma.202005423. 33118265

[ref67] Cao F.; Zhang L.; Wang H.; You Y.; Wang Y.; Gao N.; Ren J.; Qu X. Defect-rich adhesive nanozymes as efficient antibiotics for enhanced bacterial inhibition. Angewandte Chemie International Edition. 2019, 58 (45), 16236–42. 10.1002/anie.201908289. 31456332

[ref68] Shan J.; Yang K.; Xiu W.; Qiu Q.; Dai S.; Yuwen L.; Weng L.; Teng Z.; Wang L. Cu2MoS4 Nanozyme with NIR-II Light Enhanced Catalytic Activity for Efficient Eradication of Multidrug-Resistant Bacteria. Small. 2020, 16 (40), 2001099 10.1002/smll.202001099. 32893455

[ref69] Wei F.; Cui X.; Wang Z.; Dong C.; Li J.; Han X. Recoverable peroxidase-like Fe3O4@ MoS2-Ag nanozyme with enhanced antibacterial ability. Chemical Engineering Journal. 2021, 408, 127240 10.1016/j.cej.2020.127240. 33052192 PMC7536174

[ref70] Liu Z.; Wang F.; Ren J.; Qu X. A series of MOF/Ce-based nanozymes with dual enzyme-like activity disrupting biofilms and hindering recolonization of bacteria. Biomaterials. 2019, 208, 21–31. 10.1016/j.biomaterials.2019.04.007. 30986610

[ref71] Zhang L.; Liu Z.; Deng Q.; Sang Y.; Dong K.; Ren J.; Qu X. Nature-inspired construction of MOF@ COF nanozyme with active sites in tailored microenvironment and pseudopodia-like surface for enhanced bacterial inhibition. Angewandte Chemie International Edition. 2021, 60 (7), 3469–74. 10.1002/anie.202012487. 33118263

[ref72] Jiao L.; Yan H.; Wu Y.; Gu W.; Zhu C.; Du D.; Lin Y. When nanozymes meet single-atom catalysis. Angew. Chem. 2020, 132 (7), 2585–96. 10.1002/ange.201905645. 31209985

[ref73] Huang L.; Chen J.; Gan L.; Wang J.; Dong S. Single-atom nanozymes. Sci. Adv. 2019, 5 (5), eaav5490 10.1126/sciadv.aav5490. 31058221 PMC6499548

[ref74] Yan R.; Sun S.; Yang J.; Long W.; Wang J.; Mu X.; Li Q.; Hao W.; Zhang S.; Liu H.; Gao Y.; Ouyang L.; Chen J.; Liu S.; Zhang X.-D.; Ming D. Nanozyme-based bandage with single-atom catalysis for brain trauma. ACS Nano 2019, 13 (10), 11552–60. 10.1021/acsnano.9b05075. 31553878

[ref75] Shi X.; Yang J.; Wen X.; Tian F.; Li C. Oxygen vacancy enhanced biomimetic superoxide dismutase activity of CeO2-Gd nanozymes. Journal of Rare Earths. 2021, 39 (9), 1108–16. 10.1016/j.jre.2020.06.019.

[ref76] Liu Y.; Yao M.; Han W.; Zhang H.; Zhang S. Construction of a Single-Atom Nanozyme for Enhanced Chemodynamic Therapy and Chemotherapy. Chemistry–A. European Journal. 2021, 27 (53), 13418–25. 10.1002/chem.202102016. 34263950

[ref77] Pramanik A.; Laha D.; Bhattacharya D.; Pramanik P.; Karmakar P. A novel study of antibacterial activity of copper iodide nanoparticle mediated by DNA and membrane damage. Colloids Surf. B Biointerfaces. 2012, 96, 50–5. 10.1016/j.colsurfb.2012.03.021. 22521682

[ref78] Chatterjee A. K.; Chakraborty R.; Basu T. Mechanism of antibacterial activity of copper nanoparticles. Nanotechnology. 2014, 25 (13), 135101 10.1088/0957-4484/25/13/135101. 24584282

[ref79] Slavin Y. N.; Asnis J.; Häfeli U. O.; Bach H. Metal nanoparticles: Understanding the mechanisms behind antibacterial activity. Journal of Nanobiotechnology. 2017, 15 (1), 65 10.1186/s12951-017-0308-z. 28974225 PMC5627441

[ref80] Thomas-Moore B. A.; Del Valle C. A.; Field R. A.; Marin M. J. Recent advances in nanoparticle-based targeting tactics for antibacterial photodynamic therapy. Photochem. Photobiol. Sci. 2022, 21 (6), 1111–31. 10.1007/s43630-022-00194-3. 35384638 PMC9287206

[ref81] Rutkowski M.; Krzemińska-Fiedorowicz L.; Khachatryan G.; Kabacińska J.; Tischner M.; Suder A.; Kulik K.; Lenart-Boroń A. Antibacterial Properties of Biodegradable Silver Nanoparticle Foils Based on Various Strains of Pathogenic Bacteria Isolated from the Oral Cavity of Cats, Dogs and Horses. Materials. 2022, 15 (3), 1269 10.3390/ma15031269. 35161213 PMC8840282

[ref82] Alshammari F.; Alshammari B.; Moin A.; Alamri A.; Al Hagbani T.; Alobaida A.; Baker A.; Khan S.; Rizvi S. M. Ceftriaxone mediated synthesized gold nanoparticles: a nano-therapeutic tool to target bacterial resistance. Pharmaceutics. 2021, 13 (11), 1896 10.3390/pharmaceutics13111896. 34834310 PMC8622407

[ref83] Shamaila S.; Zafar N.; Riaz S.; Sharif R.; Nazir J.; Naseem S. Gold nanoparticles: An efficient antimicrobial agent against enteric bacterial human pathogen. Nanomaterials. 2016, 6 (4), 71 10.3390/nano6040071. 28335198 PMC5302575

[ref84] Zhao Y.; Tian Y.; Cui Y.; Liu W.; Ma W.; Jiang X. Small molecule-capped gold nanoparticles as potent antibacterial agents that target gram-negative bacteria. J. Am. Chem. Soc. 2010, 132 (35), 12349–56. 10.1021/ja1028843. 20707350

[ref85] Ashmore D. A.; Chaudhari A.; Barlow B.; Barlow B.; Harper T.; Vig K.; Miller M.; Singh S.; Nelson E.; Pillai S. Evaluation of E. coli inhibition by plain and polymer-coated silver nanoparticles. Rev. Inst. Med. Trop. Sao Paulo 2018, 60, e18 10.1590/s1678-9946201860018. 29694600 PMC5956551

[ref86] Nogueira S. S.; de Araujo-Nobre A. R.; Mafud A. C.; Guimarães M. A.; Alves M. M. M.; Placido A.; Carvalho F. A. A.; Arcanjo D. D. R.; Mascarenhas Y.; Costa F. G.; Albuquerque P.; Eaton P.; de Souza de Almeida Leite J. R.; da Silva D. A.; Cardoso V. S. Silver nanoparticle stabilized by hydrolyzed collagen and natural polymers: Synthesis, characterization and antibacterial-antifungal evaluation. International journal of biological macromolecules. 2019, 135, 808–14. 10.1016/j.ijbiomac.2019.05.214. 31158421

[ref87] Negi A.; Kesari K. K. Chitosan Nanoparticle Encapsulation of Antibacterial Essential Oils. Micromachines. 2022, 13 (8), 1265 10.3390/mi13081265. 36014186 PMC9415589

[ref88] Lu Y.; Feng N.; Du Y.; Yu R. Nanoparticle-Based Therapeutics to Overcome Obstacles in the Tumor Microenvironment of Hepatocellular Carcinoma. Nanomaterials (Basel). 2022, 12 (16), 2832 10.3390/nano12162832. 36014696 PMC9414814

[ref89] Espeche Turbay M. B.; Rey V.; Dorado R. D.; Sosa M. C.; Borsarelli C. D. Silver nanoparticle-protein interactions and the role of lysozyme as an antagonistic antibacterial agent. Colloids Surf. B Biointerfaces. 2021, 208, 112030 10.1016/j.colsurfb.2021.112030. 34419807

[ref90] Gehring J.; Trepka B.; Klinkenberg N.; Bronner H.; Schleheck D.; Polarz S. Sunlight-Triggered Nanoparticle Synergy: Teamwork of Reactive Oxygen Species and Nitric Oxide Released from Mesoporous Organosilica with Advanced Antibacterial Activity. J. Am. Chem. Soc. 2016, 138 (9), 3076–84. 10.1021/jacs.5b12073. 26883897

[ref91] Oves M.; Rauf M. A.; Hussain A.; Qari H. A.; Khan A. A.; Muhammad P.; Rehman M. T.; Alajmi M. F.; Ismail I. I. Antibacterial silver nanomaterial synthesis from Mesoflavibacter zeaxanthinifaciens and targeting biofilm formation. Frontiers in pharmacology. 2019, 10, 801 10.3389/fphar.2019.00801. 31427961 PMC6688106

[ref92] Ananda A. P.; Manukumar H. M.; Krishnamurthy N. B.; Nagendra B. S.; Savitha K. R. Assessment of antibacterial efficacy of a biocompatible nanoparticle PC@AgNPs against Staphylococcus aureus. Microb Pathog. 2019, 126, 27–39. 10.1016/j.micpath.2018.10.029. 30366128

[ref93] Lemire J. A.; Harrison J. J.; Turner R. J. Antimicrobial activity of metals: Mechanisms, molecular targets and applications. Nature Reviews Microbiology. 2013, 11 (6), 371–84. 10.1038/nrmicro3028. 23669886

[ref94] Tao Y.; Ju E.; Ren J.; Qu X. Bifunctionalized mesoporous silica-supported gold nanoparticles: Intrinsic oxidase and peroxidase catalytic activities for antibacterial applications. Adv. Mater. 2015, 27 (6), 1097–104. 10.1002/adma.201405105. 25655182

[ref95] Wang Y.; Ding X.; Chen Y.; Guo M.; Zhang Y.; Guo X.; Gu H. Antibiotic-loaded, silver core-embedded mesoporous silica nanovehicles as a synergistic antibacterial agent for the treatment of drug-resistant infections. Biomaterials. 2016, 101, 207–16. 10.1016/j.biomaterials.2016.06.004. 27294538

[ref96] Costas-Selas C.; Martinez-Garcia S.; Logares R.; Hernandez-Ruiz M.; Teira E. Role of Bacterial Community Composition as a Driver of the Small-Sized Phytoplankton Community Structure in a Productive Coastal System. Microb Ecol. [Online early access], 2022, 1–18, 10.1007/s00248-022-02125-2. PMC10335964 36305941

[ref97] Mortazavi V.; Nahrkhalaji M. M.; Fathi M. H.; Mousavi S. B.; Esfahani B. N. Antibacterial effects of sol-gel-derived bioactive glass nanoparticle on aerobic bacteria. J. Biomed Mater. Res. A 2010, 94A (1), 160–168. 10.1002/jbm.a.32678. 20127997

[ref98] Evliyaoglu Y.; Kobaner M.; Celebi H.; Yelsel K.; Dogan A. The efficacy of a novel antibacterial hydroxyapatite nanoparticle-coated indwelling urinary catheter in preventing biofilm formation and catheter-associated urinary tract infection in rabbits. Urol Res. 2011, 39 (6), 443–9. 10.1007/s00240-011-0379-5. 21484419

[ref99] Wei J.; Guo-Wang P.; Han Q.; Ding J.; Chen X. Preparation of antibacterial silver nanoparticle-coated PLLA grafted hydroxyapatite/PLLA composite electrospun fiber. J. Controlled Release 2015, 213, e62–3. 10.1016/j.jconrel.2015.05.103. 27005202

[ref100] Sanna V.; Sechi M. Nanoparticle therapeutics for prostate cancer treatment. Nanomedicine. 2012, 8 (1), S31–6. 10.1016/j.nano.2012.05.009. 22640911

[ref101] Nederberg F.; Zhang Y.; Tan J. P. K.; Xu K.; Wang H.; Yang C.; Gao S.; Guo X. D.; Fukushima K.; Li L.; Hedrick J. L.; Yang Y.-Y. Biodegradable nanostructures with selective lysis of microbial membranes. Nature chemistry. 2011, 3 (5), 409–414. 10.1038/nchem.1012. 21505501

[ref102] Wang X.; Liu X.; Han H. Evaluation of antibacterial effects of carbon nanomaterials against copper-resistant Ralstonia solanacearum. Colloids and Surfaces B: Biointerfaces. 2013, 103, 136–42. 10.1016/j.colsurfb.2012.09.044. 23201730

[ref103] Dizaj S. M.; Lotfipour F.; Barzegar-Jalali M.; Zarrintan M. H.; Adibkia K. Antimicrobial activity of the metals and metal oxide nanoparticles. Materials Science and Engineering: C 2014, 44, 278–84. 10.1016/j.msec.2014.08.031. 25280707

[ref104] Rice K. C.; Bayles K. W. Molecular control of bacterial death and lysis. Microbiology and molecular biology reviews. 2008, 72 (1), 85–109. 10.1128/MMBR.00030-07. 18322035 PMC2268280

[ref105] Thill A.; Zeyons O.; Spalla O.; Chauvat F.; Rose J.; Auffan M.; Flank A. M. Cytotoxicity of CeO2 nanoparticles for Escherichia coli. Physico-chemical insight of the cytotoxicity mechanism. Environmental science & technology. 2006, 40 (19), 6151–6. 10.1021/es060999b. 17051814

[ref106] Ramalingam B.; Parandhaman T.; Das S. K. Antibacterial effects of biosynthesized silver nanoparticles on surface ultrastructure and nanomechanical properties of gram-negative bacteria viz. Escherichia coli and Pseudomonas aeruginosa. ACS applied materials & interfaces. 2016, 8 (7), 4963–76. 10.1021/acsami.6b00161. 26829373

[ref107] Punniyakotti P.; Panneerselvam P.; Perumal D.; Aruliah R.; Angaiah S. Anti-bacterial and anti-biofilm properties of green synthesized copper nanoparticles from Cardiospermum halicacabum leaf extract. Bioprocess Biosyst Eng. 2020, 43 (9), 1649–57. 10.1007/s00449-020-02357-x. 32367495

[ref108] Huang X.; Bao X.; Liu Y.; Wang Z.; Hu Q. Catechol-Functional Chitosan/Silver Nanoparticle Composite as a Highly Effective Antibacterial Agent with Species-Specific Mechanisms. Sci. Rep. 2017, 7 (1), 1860 10.1038/s41598-017-02008-4. 28500325 PMC5431845

[ref109] Ninganagouda S.; Rathod V.; Singh D.; Hiremath J.; Singh A. K.; Mathew J. Growth kinetics and mechanistic action of reactive oxygen species released by silver nanoparticles from Aspergillus niger on Escherichia coli. Biomed Res. Int. 2014, 2014, 753419 10.1155/2014/753419. 25028666 PMC4083831

[ref110] Leung Y. H.; Ng A. M. C.; Xu X.; Shen Z.; Gethings L. A.; Wong M. T.; Chan C. M. N.; Guo M. Y.; Ng Y. H.; Djurisic A. B.; Lee P. K. H.; Chan W. K.; Yu L. H.; Phillips D. L.; Ma A. P. Y.; Leung F. C. C. Mechanisms of antibacterial activity of MgO: non-ROS mediated toxicity of MgO nanoparticles towards Escherichia coli. Small. 2014, 10 (6), 1171–1183. 10.1002/smll.201302434. 24344000

[ref111] Pan X.; Wang Y.; Chen Z.; Pan D.; Cheng Y.; Liu Z.; Lin Z.; Guan X. Investigation of antibacterial activity and related mechanism of a series of nano-Mg (OH) 2. ACS applied materials & interfaces. 2013, 5 (3), 1137–42. 10.1021/am302910q. 23301496

[ref112] Mukha I. P.; Eremenko A. M.; Smirnova N. P.; Mikhienkova A. I.; Korchak G. I.; Gorchev V. F.; Chunikhin A. Y. Antimicrobial activity of stable silver nanoparticles of a certain size. Applied biochemistry and microbiology. 2013, 49, 199–206. 10.1134/S0003683813020117. 23795483

[ref113] Dong Y.; Zhu H.; Shen Y.; Zhang W.; Zhang L. Antibacterial activity of silver nanoparticles of different particle size against Vibrio Natriegens. PloS one. 2019, 14 (9), e0222322 10.1371/journal.pone.0222322. 31518380 PMC6743781

[ref114] Oves M.; Rauf M. A.; Hussain A.; Qari H. A.; Khan A. A.; Muhammad P.; Rehman M. T.; Alajmi M. F.; Ismail I. I. Antibacterial silver nanomaterial synthesis from Mesoflavibacter zeaxanthinifaciens and targeting biofilm formation. Frontiers in pharmacology. 2019, 10, 801 10.3389/fphar.2019.00801. 31427961 PMC6688106

[ref115] Cui Y.; Zhao Y.; Tian Y.; Zhang W.; Lü X.; Jiang X. The molecular mechanism of action of bactericidal gold nanoparticles on Escherichia coli. Biomaterials. 2012, 33 (7), 2327–33. 10.1016/j.biomaterials.2011.11.057. 22182745

[ref116] Ventola C. L. Progress in nanomedicine: Approved and investigational nanodrugs. P and T 2017, 42 (12), 742–755. 29234213 PMC5720487

[ref117] Sharma S. K.; Sharma A. R.; Pamidimarri S. D.; Gaur J.; Singh B. P.; Sekar S.; Kim D. Y.; Lee S. S. Bacterial compatibility/toxicity of biogenic silica (b-SiO2) nanoparticles synthesized from biomass rice husk ash. Nanomaterials. 2019, 9 (10), 1440 10.3390/nano9101440. 31614501 PMC6835479

[ref118] Perera W.; Dissanayake D.; Unagolla J. M.; De Silva R. T.; Bathige S.; Pahalagedara L. R. Albumin grafted coaxial electrosparyed polycaprolactone-zinc oxide nanoparticle for sustained release and activity enhanced antibacterial drug delivery. RSC Adv. 2022, 12 (3), 1718–27. 10.1039/D1RA07847J. 35425191 PMC8978970

[ref119] Wang W. B.; Clapper J. C. Antibacterial Activity of Electrospun Polyacrylonitrile Copper Nanoparticle Nanofibers on Antibiotic Resistant Pathogens and Methicillin Resistant Staphylococcus aureus (MRSA). Nanomaterials (Basel). 2022, 12 (13), 2139 10.3390/nano12132139. 35807975 PMC9268565

[ref120] Abdelghany S. M.; Quinn D. J.; Ingram R. J.; Gilmore B. F.; Donnelly R. F.; Taggart C. C.; Scott C. J. Gentamicin-loaded nanoparticles show improved antimicrobial effects towards Pseudomonas aeruginosa infection. Int. J. Nanomedicine 2012, 7, 4053–4063. 10.2147/IJN.S34341. 22915848 PMC3418173

[ref121] Brown A. N.; Smith K.; Samuels T. A.; Lu J.; Obare S. O.; Scott M. E. Nanoparticles functionalized with ampicillin destroy multiple-antibiotic-resistant isolates of Pseudomonas aeruginosa and Enterobacter aerogenes and methicillin-resistant Staphylococcus aureus. Appl. Environ. Microbiol. 2012, 78 (8), 2768–74. 10.1128/AEM.06513-11. 22286985 PMC3318834

[ref122] Li C. H.; Chen X.; Landis R. F.; Geng Y.; Makabenta J. M.; Lemnios W.; Gupta A.; Rotello V. M. Phytochemical-Based Nanocomposites for the Treatment of Bacterial Biofilms. ACS Infectious Diseases. 2019, 5 (9), 1590–6. 10.1021/acsinfecdis.9b00134. 31251554 PMC8559558

[ref123] Kalhapure R. S.; Jadhav M.; Rambharose S.; Mocktar C.; Singh S.; Renukuntla J.; Govender T. pH-responsive chitosan nanoparticles from a novel twin-chain anionic amphiphile for controlled and targeted delivery of vancomycin. Colloids and surfaces b: biointerfaces. 2017, 158, 650–7. 10.1016/j.colsurfb.2017.07.049. 28763772

[ref124] Wang S. G.; Chen Y. C.; Chen Y. C. Antibacterial gold nanoparticle-based photothermal killing of vancomycin-resistant bacteria. Nanomedicine (Lond). 2018, 13 (12), 1405–16. 10.2217/nnm-2017-0380. 29972649

[ref125] Wu Y.; Song Z.; Wang H.; Han H. Endogenous stimulus-powered antibiotic release from nanoreactors for a combination therapy of bacterial infections. Nature Communications. 2019, 10 (1), 4464 10.1038/s41467-019-12233-2. PMC6775118 31578336

[ref126] Mulani M. S.; Kamble E. E.; Kumkar S. N.; Tawre M. S.; Pardesi K. R. Emerging strategies to combat ESKAPE pathogens in the era of antimicrobial resistance: A review. Frontiers in Microbiology. 2019, 10, 1 10.3389/fmicb.2019.00539. 30988669 PMC6452778

[ref127] Hsu C. Y.; Yang S. C.; Sung C. T.; Weng Y. H.; Fang J. Y. Anti-MRSA malleable liposomes carrying chloramphenicol for ameliorating hair follicle targeting. International Journal of Nanomedicine. 2017, 12, 8227–38. 10.2147/IJN.S147226. 29184410 PMC5689027

[ref128] Skariyachan S.; Parveen A.; Garka S. Nanoparticle Fullerene (C60) demonstrated stable binding with antibacterial potential towards probable targets of drug resistant Salmonella typhi - a computational perspective and in vitro investigation. J. Biomol Struct Dyn. 2017, 35 (16), 3449–68. 10.1080/07391102.2016.1257441. 27817242

[ref129] Ibarra J. A.; Steele-Mortimer O. Salmonella - the ultimate insider. Salmonella virulence factors that modulate intracellular survival. Cellular Microbiology. 2009, 11 (11), 1579–86. 10.1111/j.1462-5822.2009.01368.x. 19775254 PMC2774479

[ref130] Xie S.; Yang F.; Tao Y.; Chen D.; Qu W.; Huang L.; Liu Z.; Pan Y.; Yuan Z. Enhanced intracellular delivery and antibacterial efficacy of enrofloxacin-loaded docosanoic acid solid lipid nanoparticles against intracellular Salmonella. Scientific reports. 2017, 7 (1), 41104. 28112240 10.1038/srep41104 PMC5253767

[ref131] Menina S.; Eisenbeis J.; Kamal M. A.; Koch M.; Bischoff M.; Gordon S.; Loretz B.; Lehr C. M. Bioinspired liposomes for oral delivery of colistin to combat intracellular infections by Salmonella enterica. Advanced healthcare materials. 2019, 8 (17), 1900564 10.1002/adhm.201900564. 31328434

[ref132] Russell D. G. Mycobacterium tuberculosis: Here today, and here tomorrow. Nature Reviews Molecular Cell Biology. 2001, 2 (8), 569–77. 10.1038/35085034. 11483990

[ref133] Ellis T.; Chiappi M.; Garcia-Trenco A.; Al-Ejji M.; Sarkar S.; Georgiou T. K.; Shaffer M. S.; Tetley T. D.; Schwander S.; Ryan M. P.; Porter A. E. Multimetallic microparticles increase the potency of rifampicin against intracellular Mycobacterium tuberculosis. ACS Nano 2018, 12 (6), 5228–40. 10.1021/acsnano.7b08264. 29767993

[ref134] Tomasi F. G.; Rubin E. J. Failing upwards: Genetics-based strategies to improve antibiotic discovery and efficacy in Mycobacterium tuberculosis. Front Cell Infect Microbiol. 2022, 12, 932556 10.3389/fcimb.2022.932556. 36189351 PMC9519881

[ref135] Lee B.; Park J.; Ryu M.; Kim S.; Joo M.; Yeom J. H.; Kim S.; Park Y.; Lee K.; Bae J. Antimicrobial peptide-loaded gold nanoparticle-DNA aptamer conjugates as highly effective antibacterial therapeutics against Vibrio vulnificus. Scientific reports. 2017, 7 (1), 13572 10.1038/s41598-017-14127-z. 29051620 PMC5648795

[ref136] Yeom J. H.; Lee B.; Kim D.; Lee J. K.; Kim S.; Bae J.; Park Y.; Lee K. Gold nanoparticle-DNA aptamer conjugate-assisted delivery of antimicrobial peptide effectively eliminates intracellular Salmonella enterica serovar Typhimurium. Biomaterials. 2016, 104, 43–51. 10.1016/j.biomaterials.2016.07.009. 27424215

[ref137] Wu J.; Li F.; Hu X.; Lu J.; Sun X.; Gao J.; Ling D. Responsive assembly of silver nanoclusters with a biofilm locally amplified bactericidal effect to enhance treatments against multi-drug-resistant bacterial infections. ACS central science. 2019, 5 (8), 1366–76. 10.1021/acscentsci.9b00359. 31482119 PMC6716126

[ref138] Mahmoudi M.; Serpooshan V. Silver-coated engineered magnetic nanoparticles are promising for the success in the fight against antibacterial resistance threat. ACS Nano 2012, 6 (3), 2656–64. 10.1021/nn300042m. 22397679

[ref139] Meers P.; Neville M.; Malinin V.; Scotto A. W.; Sardaryan G.; Kurumunda R.; Mackinson C.; James G.; Fisher S.; Perkins W. R. Biofilm penetration, triggered release and in vivo activity of inhaled liposomal amikacin in chronic Pseudomonas aeruginosa lung infections. J. Antimicrob. Chemother. 2008, 61 (4), 859–68. 10.1093/jac/dkn059. 18305202

[ref140] Baelo A.; Levato R.; Julián E.; Crespo A.; Astola J.; Gavaldà J.; Engel E.; Mateos-Timoneda M. A.; Torrents E. Disassembling bacterial extracellular matrix with DNase-coated nanoparticles to enhance antibiotic delivery in biofilm infections. J. Controlled Release 2015, 209, 150–8. 10.1016/j.jconrel.2015.04.028. 25913364

[ref141] Habimana O.; Zanoni M.; Vitale S.; O’Neill T.; Scholz D.; Xu B.; Casey E. One particle, two targets: a combined action of functionalised gold nanoparticles, against Pseudomonas fluorescens biofilms. Journal of colloid and interface science. 2018, 526, 419–28. 10.1016/j.jcis.2018.05.014. 29763820

[ref142] Li J.; Nickel R.; Wu J.; Lin F.; van Lierop J.; Liu S. A new tool to attack biofilms: driving magnetic iron-oxide nanoparticles to disrupt the matrix. Nanoscale. 2019, 11 (14), 6905–15. 10.1039/C8NR09802F. 30912773

[ref143] Rabin N.; Zheng Y.; Opoku-Temeng C.; Du Y.; Bonsu E.; Sintim H. O. Biofilm formation mechanisms and targets for developing antibiofilm agents. Future Med. Chem. 2015, 7 (4), 493–512. 10.4155/fmc.15.6. 25875875

[ref144] Miller K. P.; Wang L.; Chen Y. P.; Pellechia P. J.; Benicewicz B. C.; Decho A. W. Engineering nanoparticles to silence bacterial communication. Front Microbiol. 2015, 6, 189 10.3389/fmicb.2015.00189. 25806030 PMC4354405

[ref145] Eifler A. C.; Thaxton C. S. Nanoparticle therapeutics: FDA approval, clinical trials, regulatory pathways, and case study. Methods Mol. Biol. 2011, 726, 325–38. 10.1007/978-1-61779-052-2_21. 21424459

[ref146] Kotta M.; Gorantla S.; Muddada V.; Lenka R. R.; Karri T.; Kumar S.; Tivanani M. Antibacterial activity and debonding force of different lingual retainers bonded with conventional composite and nanoparticle containing composite: An in vitro study. Journal of the World Federation of Orthodontists. 2020, 9 (2), 80–5. 10.1016/j.ejwf.2020.03.001. 32672659

[ref147] Zhou Z.; Hu F.; Hu S.; Kong M.; Feng C.; Liu Y.; Cheng X.; Ji Q.; Chen X. pH-Activated nanoparticles with targeting for the treatment of oral plaque biofilm. J. Mater. Chem. B 2018, 6 (4), 586–92. 10.1039/C7TB02682J. 32254487

[ref148] Chen S.; Xu X. L.; Zhou B.; Tian J.; Luo B. M.; Zhang L. M. Acidic pH-Activated Gas-Generating Nanoparticles with Pullulan Decorating for Hepatoma-Targeted Ultrasound Imaging. ACS Appl. Mater. Interfaces. 2019, 11 (25), 22194–205. 10.1021/acsami.9b06745. 31199110

[ref149] Zhou J.; Horev B.; Hwang G.; Klein M. I.; Koo H.; Benoit D. S. Characterization and optimization of pH-responsive polymer nanoparticles for drug delivery to oral biofilms. J. Mater. Chem. B 2016, 4 (18), 3075–85. 10.1039/C5TB02054A. 27429754 PMC4943847

[ref150] Gao L.; Liu Y.; Kim D.; Li Y.; Hwang G.; Naha P. C.; Cormode D. P.; Koo H. Nanocatalysts promote Streptococcus mutans biofilm matrix degradation and enhance bacterial killing to suppress dental caries in vivo. Biomaterials. 2016, 101, 272–84. 10.1016/j.biomaterials.2016.05.051. 27294544 PMC4949957

[ref151] Naha P. C.; Liu Y.; Hwang G.; Huang Y.; Gubara S.; Jonnakuti V.; Simon-Soro A.; Kim D.; Gao L.; Koo H.; Cormode D. P. Dextran-coated iron oxide nanoparticles as biomimetic catalysts for localized and pH-activated biofilm disruption. ACS Nano 2019, 13 (5), 4960–71. 10.1021/acsnano.8b08702. 30642159 PMC7059368

[ref152] Liu Y.; Naha P. C.; Hwang G.; Kim D.; Huang Y.; Simon-Soro A.; Jung H.-I.; Ren Z.; Li Y.; Gubara S.; Alawi F.; Zero D.; Hara A. T.; Cormode D. P.; Koo H. Topical ferumoxytol nanoparticles disrupt biofilms and prevent tooth decay in vivo via intrinsic catalytic activity. Nature communications. 2018, 9, 2920 10.1038/s41467-018-05342-x. PMC6068184 30065293

[ref153] Masoud S. S.; Kovacevich A.; Gangji R.; Nyawale H.; Nyange M.; Ntukula A. Extent and Resistance Patterns of ESKAPE Pathogens Isolated in Pus Swabs from Hospitalized Patients. Can. J. Infect Dis Med. Microbiol. 2022, 2022, 3511306 10.1155/2022/3511306. 36353409 PMC9640227

[ref154] Tang J.; Liu X.; Ge Y.; Wang F. Silver Nanoparticle-Anchored Human Hair Kerateine/PEO/PVA Nanofibers for Antibacterial Application and Cell Proliferation. Molecules. 2021, 26 (9), 2783 10.3390/molecules26092783. 34066875 PMC8125921

[ref155] Ahire J. J.; Hattingh M.; Neveling D. P.; Dicks L. M. Copper-Containing Anti-Biofilm Nanofiber Scaffolds as a Wound Dressing Material. PLoS One. 2016, 11 (3), e0152755 10.1371/journal.pone.0152755. 27028292 PMC4814046

[ref156] Liu C.; Yao W.; Tian M.; Wei J.; Song Q.; Qiao W. Mussel-inspired degradable antibacterial polydopamine/silica nanoparticle for rapid hemostasis. Biomaterials. 2018, 179, 83–95. 10.1016/j.biomaterials.2018.06.037. 29980077

[ref157] Wang J.; Chen X.-Y.; Zhao Y.; Yang Y.; Wang W.; Wu C.; Yang B.; Zhang Z.; Zhang L.; Liu Y.; Du X.; Li W.; Qiu L.; Jiang P.; Mou X.-Z.; Li Y.-Q. pH-switchable antimicrobial nanofiber networks of hydrogel eradicate biofilm and rescue stalled healing in chronic wounds. ACS Nano 2019, 13 (10), 11686–11697. 10.1021/acsnano.9b05608. 31490650

[ref158] Diez-Pascual A. M. Antibacterial Action of Nanoparticle Loaded Nanocomposites Based on Graphene and Its Derivatives: A Mini-Review. Int. J. Mol. Sci. 2020, 21 (10), 3563 10.3390/ijms21103563. 32443558 PMC7278957

[ref159] Hettiarachchi S. D.; Zhou Y.; Seven E.; Lakshmana M. K.; Kaushik A. K.; Chand H. S.; Leblanc R. M. Nanoparticle-mediated approaches for Alzheimer’s disease pathogenesis, diagnosis, and therapeutics. Journal of controlled release. 2019, 314, 125–40. 10.1016/j.jconrel.2019.10.034. 31647979

[ref160] Ahmed T.; Shahid M.; Noman M.; Niazi M. B.; Mahmood F.; Manzoor I.; Zhang Y.; Li B.; Yang Y.; Yan C.; Chen J. Silver nanoparticles synthesized by using Bacillus cereus SZT1 ameliorated the damage of bacterial leaf blight pathogen in rice. Pathogens. 2020, 9 (3), 160 10.3390/pathogens9030160. 32110981 PMC7157244

[ref161] Ratih D. N.; Mulyawati E.; Santi R. K.; Kristanti Y. Antibacterial and Cytotoxicity of Root Canal Sealer with the Addition of Chitosan Nanoparticle at Various Concentrations.. Eur. J. Dent. 2022, 2022, 1 10.1055/s-0042-1746415. PMC10329522 35728614

[ref162] Hossen N.; Kajimoto K.; Akita H.; Hyodo M.; Harashima H. A comparative study between nanoparticle-targeted therapeutics and bioconjugates as obesity medication. J. Controlled Release 2013, 171 (2), 104–12. 10.1016/j.jconrel.2013.07.013. 23871959

[ref163] Xi J.; Wu Q.; Xu Z.; Wang Y.; Zhu B.; Fan L.; Gao L. Aloe-emodin/carbon nanoparticle hybrid gels with light-induced and long-term antibacterial activity. ACS Biomaterials Science & Engineering. 2018, 4 (12), 4391–400. 10.1021/acsbiomaterials.8b00972. 33418832

[ref164] Xiao W.; Xu J.; Liu X.; Hu Q.; Huang J. Antibacterial hybrid materials fabricated by nanocoating of microfibril bundles of cellulose substance with titania/chitosan/silver-nanoparticle composite films. J. Mater. Chem. B 2013, 1 (28), 3477–85. 10.1039/c3tb20303d. 32260939

[ref165] Kim D. H.; Kim K. H.; Kwon T. Y.; Choi S. H.; Kang S. S.; Kwon S. T.; Cho D. H.; Kim H. D.; Son J. S. Antibacterial releasing titanium surface using albumin nanoparticle carriers. Journal of Nanoscience and Nanotechnology. 2014, 14 (11), 8422–6. 10.1166/jnn.2014.9934. 25958539

[ref166] Mohammadi-Aloucheh R.; Habibi-Yangjeh A.; Bayrami A.; Latifi-Navid S.; Asadi A. Enhanced anti-bacterial activities of ZnO nanoparticles and ZnO/CuO nanocomposites synthesized using Vaccinium arctostaphylos L. fruit extract. Artif Cells Nanomed. Biotechnol. 2018, 46 (1), 1200–9. 10.1080/21691401.2018.1448988. 29527924

[ref167] Deokar A. R.; Perelshtein I.; Saibene M.; Perkas N.; Mantecca P.; Nitzan Y.; Gedanken A. Antibacterial and in vivo studies of a green, one-pot preparation of copper/zinc oxide nanoparticle-coated bandages. Membranes. 2021, 11 (7), 462 10.3390/membranes11070462. 34206493 PMC8305234

[ref168] Dong S.; Hua H.; Wu X.; Mao X.; Li N.; Zhang X.; Wang K.; Yang S. In-situ photoreduction strategy for synthesis of silver nanoparticle-loaded PVDF ultrafiltration membrane with high antibacterial performance and stability. Environmental Science and Pollution Research. 2023, 30, 26445 10.1007/s11356-022-24052-y. 36369440

[ref169] Patil M. P.; Seo Y. B.; Kim G. D. Morphological changes of bacterial cells upon exposure of silver-silver chloride nanoparticles synthesized using Agrimonia pilosa. Microb Pathog. 2018, 116, 84–90. 10.1016/j.micpath.2018.01.018. 29339306

[ref170] Bharadwaj V. N.; Nguyen D. T.; Kodibagkar V. D.; Stabenfeldt S. E. Nanoparticle-Based Therapeutics for Brain Injury. Adv. Healthc Mater. 2018, 7 (1), 1700668 10.1002/adhm.201700668. PMC5903677 29034608

